# Rumor management in public health: a system dynamics analysis based on social trust

**DOI:** 10.3389/fpubh.2025.1713731

**Published:** 2025-12-03

**Authors:** Wei Dong, Yijie Wang, Fei Li

**Affiliations:** 1 Department of Sociology, Hohai University, Nanjing, China; 2 School of Humanities, Anhui Polytechnic University, Wuhu, China

**Keywords:** health-related rumors, public health, social trust, system dynamics, COVID-19

## Abstract

**Background:**

With the rapid development of digital society and the Internet, public health systems are increasingly confronted with novel phenomena and emergent challenges originating from cyberspace. Single medical interventions often prove insufficient to address the complex and multifaceted nature of contemporary health issues, necessitating the integration of interdisciplinary expertise.

**Methods:**

This study focuses on the propagation mechanisms of online rumors during the early stages of the COVID-19 pandemic, highlighting their intricate interactions with social trust. Using a system dynamics approach, a comprehensive “generation–diffusion–dissipation” model of rumors was constructed, revealing the differentiated role of social trust across stages.

**Results:**

Trust deficits create fertile ground for rumor emergence, while the impersonation and exploitation of trust facilitate rapid rumor diffusion beyond the bounds of rational skepticism. Conversely, trust reconstruction serves as a critical driver for rumor dissipation and the restoration of social cognition. The spread of rumors is influenced not only by information veracity but also by the embedding of social relationships, emotional mobilization, and manipulation of trust pathways.

**Conclusion:**

Effective rumor governance in public health systems requires a shift from reactive “*post hoc* debunking” toward proactive “preemptive prevention,” encompassing transparent information disclosure, interactive communication mechanisms, targeted interventions against “trust hijacking,” and trust reconstruction strategies guided by social-psychological restoration. This study employs a system dynamics approach to elucidate the mechanisms of rumor propagation while empirically validating the dynamic role of trust within the governance system. It not only offers a new analytical framework for understanding the evolution of trust under digital social conditions but also provides strategic insights for enhancing the governance capacity of public health systems in responding to rumors.

## 1 Introduction

With the rapid development of digital media, cyberspace has become a critical arena for public information exchange in contemporary society. According to the Statistical Report on China’s Internet Development, as of June 2025, the number of Internet users in China reached 1.123 billion, with an Internet penetration rate of 79.7%. The sheer scale of Internet users not only underscores the deep integration of the Internet into social life but also indicates that the scope, speed, and influence of information dissemination have reached unprecedented levels (1). Online media, through constantly evolving content and formats, has reshaped the patterns of information flow, gradually transforming social narratives from a single mainstream discourse to a diversified and coexisting ecosystem (2, 3). Against this backdrop, the dissemination of public health-related information exhibits characteristics of immediacy, wide coverage, and interactivity. At the same time, however, these conditions also provide fertile ground for the emergence and propagation of rumors and other misleading information. The empowerment of media through intelligent technologies has fundamentally reshaped both communication mechanisms and the broader information ecosystem. Rumors proliferate exponentially across various social platforms, posing new challenges to social order and governance (4).

In recent years, the scale and persistence of online rumors have become increasingly prominent. The Tencent Report on Online Rumor Governance indicates that medical and health information, food safety, and social sciences are the three most frequent domains for online rumors. On the WeChat platform alone, 17,881 rumor-debunking articles were published in a single year, reaching a total readership exceeding 114 million. Despite proactive efforts by government bodies, media outlets, and professional institutions, the phenomenon of “debunking failure” persists (5, 6). This suggests that relying solely on technological interventions and information clarification is insufficient to effectively curb rumor dissemination; the deeper underlying challenge lies in the fragility and volatility of social trust (7).

The concept of rumors has evolved in the digital era, reflecting broader public social attitudes. With the advent of risk and mediated societies, false information within the context of major risk events differs from conventional rumor studies (8). The outbreak of COVID-19 has amplified this issue. During the early stages of the pandemic, the reality of “staying at home” made online channels the primary source for public health information, with related content rapidly occupying central positions across websites and social media platforms. Simultaneously, false information associated with the pandemic spread widely within a very short time, provoking public panic and anxiety. Starting from February 2020, the Weibo platform initiated dedicated rumor-debunking efforts, delivering corrective information to users via private messages. Analyses of these cases indicate that many rumors are closely intertwined with interpersonal trust, scientific trust, and media trust, highlighting the strong interconnection between rumors and social trust and revealing how trust gaps can be exploited and amplified by false information during public health crises.

Social trust, as a fundamental pillar of societal functioning (9), plays a dual role in the diffusion of online health-related rumors. On one hand, trust deficits provide opportunities for rumor propagation, as the public is more likely to accept unverified information in the absence of reliable channels. On the other hand, the proliferation of rumors does not depend solely on the lack of trust; rather, rumors often leverage preexisting social trust networks to spread rapidly, such as through word-of-mouth among close contacts or information resharing within communities. This duality renders the governance of online health rumors highly complex. Moreover, the inherent uncertainty of an information-driven society exacerbates public risk perception and anxiety, further facilitating the psychological and social support for rumor propagation (2, 10, 11).

Existing studies on rumor governance have accumulated substantial findings, yet many focus on single technological approaches. For instance, some studies employ Matlab simulations to model the pathways and speed of rumor dissemination, aiming to enhance predictive and intervention effectiveness. However, such research often emphasizes the quantification and fitting of the propagation process, lacking a systematic explanation of the dynamic interplay between social trust and rumor dissemination. Treating social trust merely as a background variable or static condition fails to capture its evolving influence across different stages of dissemination. In reality, rumor propagation and governance constitute a dynamic feedback process: social trust can inhibit rumor generation in early stages, yet may be eroded in later stages due to governance failures, indicating the close interrelation between rumors and trust.

Within this context, system dynamics offers a novel perspective for understanding and governing online health-related rumors. By emphasizing causal feedback and temporal evolution, system dynamics characterizes complex social phenomena and reveals the interdependencies and dynamic relationships among variables. Applied to the study of health rumors in public health systems, it allows the construction of a closed-loop model linking “rumor dissemination – social trust evolution – governance feedback,” systematically illustrating the interactive logic between rumors and trust across different stages. This approach not only overcomes the limitations of traditional static analyses but also facilitates the exploration of proactive prevention and effective governance of rumors through trust mechanisms. Accordingly, this study seeks to model and simulate the interactions between social trust and rumor propagation within the context of public health systems. Specifically, it addresses the following questions: (1) What are the mechanisms through which social trust operates at different stages of online health rumor propagation? (2) How does the evolution of social trust, in turn, shape rumor dissemination pathways? (3) How can trust-based design and interventions enable preemptive prevention and effective governance of online health rumors? Through these discussions, this study aims to provide both theoretical insights and practical guidance for rumor governance in public health systems, responding to the new challenges of health risk management in digital societies.

## 2 Literature review

Rumors as a form of communication have evolved over millennia, developing alongside the dissemination of language and written texts. In cyberspace, rumors exhibit not only their intrinsic characteristics but also additional features unique to the digital environment.

### 2.1 Public health challenges of rumor dissemination

A rumor is a statement circulated informally within social groups, lacking authoritative verification, and characterized by uncertainty and ambiguity regarding its veracity (12). Scholars generally recognize that rumors are often closely linked to real-world events and enhance their credibility and transmissibility by emphasizing perceived authenticity, thereby gaining broader social recognition (13, 14). In the context of widespread Internet penetration, the primary challenge for the public has shifted from “information acquisition” to “information discernment” (2). Social media platforms, exemplified by Weibo, facilitate public communication while simultaneously accelerating the generation and dissemination of rumors. Research indicates that rumor content often exhibits high repetition, pronounced controversial viewpoints, and elevated comment and sharing rates, characteristics that contribute to viral dissemination (15–17). The decentralized nature of social media further amplifies rumor spread, making it more destructive in environments lacking regulation and countervailing credible information. Effective rumor governance therefore relies not only on individual-level cognitive interventions but also on platform-level institutional constraints and information ecosystem management. Empirical evidence linking rumor propagation rates and media environments provides justification for incorporating variables such as “propagation speed” and “platform effects” into system dynamics models (18).

From a global perspective on the social media ecosystem, the study further integrates the characteristics of platforms such as Meta and X, including open information flows, diffusion through weak ties, and algorithmically guided content recommendations. The interplay between algorithmic logic and user interaction patterns jointly shapes the distribution of nodal influence, the life cycle of information, and the dynamic evolution of social trust. Consequently, these dynamics lead to diverse rumor propagation paths and heterogeneous governance strategies. Research on misinformation in Western social media contexts has primarily focused on dissemination mechanisms, psychological drivers, and social impacts. Based on over a decade of data from the X platform, Vosoughi et al. (19) revealed that false news spreads significantly faster, farther, and deeper than true information, driven mainly by human emotions and novelty preferences rather than algorithmic manipulation. In public health contexts, Gisondi et al. (20) highlighted the detrimental effects of social media during the COVID-19 infodemic and proposed that social media platforms should collaborate with public health institutions and community influencers to improve governance.

In recent years, online rumors have become a focal point of academic research (21). Studies highlight that online rumors exhibit features such as group polarization, information cascade effects, conformity behavior, and selective assimilation of information (14). While the anonymity, interactivity, and openness of the Internet protect freedom of expression, these same characteristics facilitate the spread of rumors and false information (6). Platforms like WeChat and Weibo serve not only as communication channels but also as conduits for rapid rumor dissemination (22, 23), exposing the public to deeper risks associated with health-related misinformation even as they enjoy the conveniences of online access. Information transparency in public health governance is not merely a mechanism of information disclosure, but a fundamental institutional condition for the construction of social trust. Its core lies in promoting openness of information, transparency in decision-making, and interactive communication to reduce public uncertainty and perceived risk, thereby strengthening reliance on and trust in official information sources (24). Public health authorities can enhance transparency by regularly releasing epidemic data and prevention updates, disclosing decision-making processes and their scientific foundations, and establishing public feedback channels.

The impact of online health rumors is particularly pronounced during public health emergencies. During the COVID-19 pandemic, large segments of the population, limited by insufficient knowledge, social experience, and emotional intelligence, were vulnerable to fragmented information, leading to blind adherence to and dissemination of false information (25, 26). This phenomenon is not unprecedented: during the 2003 SARS outbreak, panic buying of Banlangen, during the 2011 Fukushima nuclear accident, panic buying of salt, and during the 2020 COVID-19 outbreak, panic buying of Shuanghuanglian, all illustrate that health-related rumors originating in virtual spaces can profoundly affect offline social life and order. Rumors thereby construct implicit linkages between virtual networks and real-world social structures, functioning as potential sources of societal risk. As a typical form of social stress response, public health crises exhibit distinctive characteristics that set them apart from crises in other sectors (27). They are marked by higher levels of suddenness and uncertainty, shorter information feedback cycles, and more frequent fluctuations in public trust. Because such crises are directly linked to individual lives and collective safety, they demonstrate heightened social sensitivity in both trust perception and policy response. The concept of an “information epidemic” emphasizes the dual threat posed by false information and rumors to public health. By influencing individual cognition and indirectly altering behavioral patterns, rumors necessitate governance strategies that go beyond information screening to include the enhancement of public health knowledge and trust at the socio-psychological level, thus providing theoretical support for subsequent system dynamics modeling, particularly regarding cognition–trust–behavior linkages (28).

Despite advancements in simulating and predicting rumor propagation with the aid of technological tools, significant gaps remain. First, most studies focus on simulation and information flow modeling, paying limited attention to the unique dissemination environment and socio-psychological context of online health rumors. Second, comprehensive interdisciplinary governance perspectives remain underdeveloped, especially regarding the central role of social trust in rumor propagation. Trust can function both as a barrier to rumor spread and, under certain conditions, be exploited by rumors to facilitate dissemination. Revealing this complex mechanism from the perspective of dynamic feedback and system interactions remains a critical research gap. The system dynamics approach has been widely applied in rumor research. Yin Fei et al. (29) employed a system dynamics model to examine the effects of media influence rates and online polarization on rumor propagation during emergencies. Li Shizheng et al. (30) constructed a rumor evolution model within mobile social networks, revealing the sensitivity of rumor diffusion to variables such as communication channels, curiosity-driven behavior, and the speed of governance response. Huo Liangan et al. (31) developed a rumor propagation dynamics model based on transmission mechanisms, verifying its stability and proposing control thresholds. Zhu et al. (32) further applied system dynamics to analyze the behavioral evolution of rumor producers, identifying negative individual emotions as a key driver in the generation of false information. Collectively, these studies demonstrate that system dynamics effectively uncovers the complex dynamic mechanisms underlying rumor propagation and provides quantitative support for the design of governance strategies.

The trust system during public health crises exhibits distinct stage-based characteristics. In the initial phase, the sudden influx of risk information leads to a sharp rise in public risk perception; in the intermediate phase, rumor propagation intertwines with the process of trust repair; and in the later phase, the trust structure is gradually reconstructed and tends toward stability. This phased evolution not only reveals the dynamic mechanisms of trust within public health crises but also reflects the social system’s capacity for self-regulation in responding to sudden public emergencies. In this context, the application of system dynamics to rumor research is of substantial significance. System dynamics emphasizes the characterization of feedback relationships and temporal evolution within complex systems, enabling a holistic understanding of the cyclical mechanism linking “rumor dissemination–social trust evolution–governance response.” This approach not only addresses shortcomings in existing research but also provides a more explanatory and practically valuable analytical framework for the governance of health-related rumors within public health systems.

### 2.2 The multidimensional mechanisms of social trust and interdisciplinary perspectives

Social trust, a central construct in the study of societal attitudes, refers to the macro-level psychological state shared by a society or its majority at a given period, which evolves in response to changes in social culture, institutional environments, and social structures (10). Social trust exists within networks of relationships at the individual, group, and even national levels, and its manifestations vary according to the social actors’ status, interests, values, lifestyles, and cultural traditions (2). From a psychological perspective, trust represents an expectation regarding others’ behavior and involves risk in future, yet-to-be-verified interactions (21). Sociology, in contrast, emphasizes the role of institutional environments, cultural norms, social networks, and resource distribution in the construction of trust. This interdisciplinary perspective helps to elucidate the multi-layered mechanisms of trust in rumor propagation, showing that trust is shaped not only by individual psychology but also by societal structures and institutional constraints.

“Social trust” possesses a multidimensional structure: macro-level trust reflects the predictability and stability of social institutions; meso-level trust concerns the relationship between authority and transparency within organizational hierarchies; and micro-level trust captures the interpersonal foundations of interaction based on experience and shared identity. This multidimensional perspective helps to explain how the decline of online trust influences offline social interactions and institutional reliance, while also revealing the potential consequences of trust erosion for public health governance (33). In cyberspace, anonymity is one of the core drivers of rapid health-related rumor dissemination, exemplifying the “stranger trust” characteristic of online societies. Luhmann’s seminal work on trust posited that trust simplifies social complexity, reduces social interaction costs, and grants familiarity and certainty to the perceived world (2). Familiar trust and stranger trust can be understood as extensions of the strong–weak tie theory, whereby the intensity of trust is positively correlated with familiarity. In online environments, due to the lack of long-term face-to-face interactions and social constraints among individuals, stranger trust becomes the norm. This trust pattern lowers the costs of rumor fabrication to some extent, facilitating rapid propagation of false information.

The formation and erosion of online trust are not isolated from offline social structures. During the pandemic, fluctuations in public trust toward various information sources reflected the legitimacy pressures faced by offline governance systems, revealing the dynamic interconnection between shifts in online trust and the underlying structures of offline society. Existing research has incorporated trust mechanisms into rumor propagation models, analyzing online dissemination dynamics from an epidemiological perspective. When information sources are highly credible, rumor diffusion slows significantly; conversely, in low-trust environments, rumors spread rapidly. Differences in trust levels not only influence propagation speed but also affect the eventual reach of rumors. Therefore, trust acts both as a suppressor of rumor spread and as a moderator of its scale. This provides theoretical justification for incorporating “social trust” as a variable in system dynamics modeling (34).

Social structure is closely related to the type of trust (9), and rumor generation often has structural social roots (21, 35). Some scholars argue that online rumors are fundamentally similar to traditional rumors, with the Internet merely accelerating dissemination (1). However, the hierarchical and differential pattern (“chaxu geju”) of social trust in China results in graded trust relationships among individuals (36). Cyberspace extends this hierarchical logic, giving rise to a novel form of trust—“network trust”—characterized by weak ties, anonymity, and cross-spatial dissemination. Network trust not only alters information flow pathways but also enables rumors to achieve wider propagation.

Comparing rumor propagation patterns in physical versus online spaces reveals the critical influence of spatial and trust relationships on dissemination. In high-trust physical spaces, social constraints and moral pressures among acquaintances raise the costs of rumor fabrication, limiting its spread; once detected, rumors can be swiftly curtailed by sanction mechanisms and social norms, making debunking relatively straightforward. In contrast, in low-trust online spaces, anonymity reduces fabrication costs, allowing rumors to spread rapidly through technological means. Their potential impact is substantial, and trust fractures in online spaces require multi-level interventions for restoration (2). Table 1 summarizes the typical rumor propagation patterns under different spatial and trust contexts.

**Table 1:** Typical rumor propagation patterns under different spatial and trust contexts.

	Real space – Strong trust	Cyberspace – Weak trust
Rumour-mongering cost	High	Low
Range of rumours	Low	High
Difficulty in refuting rumors	Low	High

This comparison indicates that the governance of online health-related rumors, characterized by weak trust, is considerably more challenging than traditional rumor management. The complexity of the governance environment increases further when social trust intersects with group interests, market competition, and organizational power (2). During the COVID-19 pandemic, high levels of trust facilitated public adherence to preventive measures and promoted cooperative behavior, whereas trust deficits were associated with reduced protective behaviors and social fragmentation. Trust thus emerges as a critical variable in pandemic governance, complementing psychological perspectives in health communication research and providing a theoretical basis for the dynamic specification of the “trust” variable in system dynamics modeling (37). Media play a multifaceted role in public health information dissemination and trust building, serving not only as information sources but also as agents of verification and clarification. Their efforts to fact-check and debunk rumors can significantly reduce public adoption of false information (38). Luo et al. (39), through a comparative analysis of English- and Chinese-language social media texts, revealed thematic differences and variations in emotional distribution of COVID-19 misinformation across linguistic and cultural contexts. Consequently, the ecology of misinformation and the corresponding model parameters across different platforms require dynamic adjustment.

The generation and dissemination of rumors are highly dynamic and nonlinear processes. Empirical studies suggest that factors such as information ambiguity, public anxiety, individual motivations, societal attention, and information transparency all influence rumor formation and propagation (26). At the same time, some rumors do not originate from a lack of trust. For example, false “emergency notices” issued under the guise of government authority reflect high public recognition of governmental credibility. “Trust hijacking,” a key concept in this study, refers to the phenomenon in which individuals or groups redirect trust—originally placed in institutions, experts, or authorities—toward unverified information sources or intermediaries. This misallocation of trust resources facilitates the spread of rumors and amplifies their impact. Accordingly, the analysis of online health rumors requires differentiated trust mechanisms, highlighting the role of varying rumor types within social-psychological, institutional, and informational contexts. Research indicates that effective communication and enhanced government credibility significantly increase public vaccination willingness and health compliance. In the absence of transparent and timely communication, rumors and misinformation are more likely to fill trust vacuums, impeding public health interventions. The effectiveness of rumor debunking is contingent upon both the transparency of governmental communication and the level of public trust (40).

In the study of rumor propagation, it is essential to consider the significant influence of underlying sociodemographic variables. Age affects the choice of information channels, patterns of internet use, and technological adaptability. Occupational type determines the frequency of exposure to public health information and reliance on trusted sources. Educational level is associated with information filtering and critical thinking skills, influencing both the ability to judge information veracity and the propensity to share it. Place of residence reflects urban–rural disparities, the speed of information circulation, and the density of social networks. While the present study’s model primarily focuses on trust variables and their dynamic feedback, the potential effects of these sociodemographic factors can enrich theoretical interpretation (41). Methodologically, qualitative research plays a crucial role in theory development and hypothesis formation. By reviewing core literature on social trust, crisis information dissemination, and rumor governance, the study distills theoretical insights relevant to model variables and feedback mechanisms, including the hierarchical structure of social trust, trust-recovery mechanisms, and the phenomenon of trust hijacking, thereby providing a clear theoretical foundation for the selection of quantitative variables and causal loops.

Within this context, system dynamics emphasizes the characterization of complex system behavior through causal loops, feedback mechanisms, and temporal evolution, enabling the simulation of interactions among rumor propagation, social trust evolution, and policy interventions. Each variable is grounded in qualitative text analysis and supported by theoretical and empirical literature. The construct of “information source credibility” draws on Lewandowsky et al. (42), who examined the relationship between trust and the cognition of false information; “network propagation intensity” is informed by Vosoughi et al. (19), who empirically analyzed the differences in the spread of false versus true information on social media; and “intervention effectiveness” is guided by Allcott et al. (43), who evaluated platform fact-checking policies and their impact on misinformation dissemination. Together, these sources establish the theoretical validity and analytical foundation of each variable (19, 42, 43). This approach reveals delay effects, cumulative impacts, and feedback cycles inherent in rumor dissemination, offering both theoretical insight and practical guidance for optimizing rumor governance strategies in public health systems across different scenarios. The integration of social trust theory with system dynamics provides a novel analytical perspective for studying online health rumors. On one hand, it explains variations in rumor propagation across different spatial and trust contexts; on the other hand, it elucidates the impact of complex feedback mechanisms on governance outcomes, thereby offering scientific support for evidence-based public health policy formulation.

## 3 Model and methods

The empirical data for this study are derived from the Weibo Rumor-Refutation database during the early outbreak of COVID-19. The collection period spans February to March 2020, a critical stage in pandemic prevention and control. The database comprises 1,100 independent observations, each corresponding to a single rumor-refutation event, thereby ensuring both temporal continuity and event completeness. The database encompasses multi-themed social media discussions, including COVID-19 situation reports and public health policies. Data were collected using keyword searches and combinatorial search strategies to identify relevant entries, with key information on debunking time, debunking agents, and debunking content extracted to construct the information diffusion network. Each observation in the database explicitly includes time stamps, account IDs, and textual content, providing essential parameters for the system dynamics model.

During the preprocessing stage, date variables were standardized to ensure comparability in time-series analysis. Source variables were classified into hierarchical categories, facilitating comparisons across different governance actors and their relative contributions to rumor-refutation activities. For text variables, we employed Chinese word segmentation and stop-word removal to reduce redundancy, followed by a hybrid approach of manual coding and automated text mining to categorize refutation content into thematic clusters. This procedure transformed unstructured textual information into analyzable structured data.

In terms of methodology, the study adopted a dual-path approach combining quantitative statistical techniques and qualitative textual analysis. Trend analysis and visualization were employed to depict the dynamic evolution of rumor-refutation activities and to identify the temporal features of rumor governance during the early pandemic stage. Additionally, high-frequency text segments were qualitatively analyzed to interpret the underlying logic of social trust and governance practices. This study employs a qualitative text analysis approach based on content analysis and thematic coding to systematically identify key concepts and themes related to public health rumor propagation and social trust. The qualitative data are drawn from analyses of specific debunking texts, news reports, and social media cases, providing insights into public trust evaluations of information sources and acceptance of interventions. This approach also helps to identify the design logic and implementation constraints of intervention strategies, while revealing rumor propagation pathways, public opinion responses, and mechanisms of network diffusion. All statistical and text processing tasks were conducted using Python and its associated libraries.

The sample selection targets active users on social networks to capture the perspectives and behaviors of different social roles in rumor governance, with a focus on social trust formation, rumor propagation pathways, and the effectiveness of intervention measures. The coding process employs thematic analysis, categorizing textual content into key concepts and patterns, thereby providing theoretical support for model variable selection, causal relationship assumptions, and the configuration of intervention scenarios. Texts were preprocessed, anonymized, and filtered for content. The analytical workflow includes reading the texts, extracting semantic units related to rumor propagation and social trust, performing open coding, and grouping themes, which are subsequently integrated into variable categories and causal relationship networks.

The Weibo network exhibits small-world characteristics and strong clustering, enabling rapid information diffusion within local groups and cross-community spread via high-influence nodes. The design of network degree distribution, average path length, and clustering coefficient provides the foundation for quantitative simulation of trust transmission and rumor diffusion mechanisms. Key variables in the model include “social trust level,” derived from coded attitudes toward information sources and operationalized using text frequency metrics; “rumor propagation rate,” measured through observed information interactions; and “intervention response effectiveness,” constructed from coded textual responses. The selection logic of each variable is distilled from qualitative thematic coding into quantifiable indicators corresponding to the parameters of the system dynamics model.

The dataset has distinct scholarly value. First, from a temporal perspective, the refutation events are highly concentrated in the outbreak’s initial phase—a period marked by acute social uncertainty and rapid rumor dissemination. This provides empirical leverage for examining the immediacy and dynamism of rumor governance during public health emergencies. Second, from the perspective of spatial coverage and governance hierarchy, the dataset includes rumor-refutation efforts by actors at national, provincial, and grassroots levels. This enables an exploration of both top-down information clarification mechanisms and bottom-up societal governance practices, offering insights into multi-level governance coordination during crises. Third, from a content perspective, the refutation themes span pandemic control, material supply, and social order. This diversity allows for comparisons across rumor categories, analyses of shifting public concerns, and investigations into the differentiated effects of trust mechanisms. Fourth, the coexistence of structured numerical variables and unstructured textual data enables multiple analytical pathways: time-series models can reveal the dynamics of rumor governance, text-mining techniques can uncover discursive patterns, and system dynamics modeling can simulate the interaction effects between social trust and rumor diffusion.

Addressing rumor governance within public health systems requires moving beyond the limitations of single-disciplinary approaches. The emergence and diffusion of online health rumors are not merely information flows in the communication sense but also encompass medical risk perception, psychological responses in emotions and behavior, mathematical modeling of dynamic processes, and the sociological mechanisms of social trust. System dynamics, with feedback loops, stock–flow structures, and time delays at its core, can capture the interactive relationships among social trust, information dissemination, and policy responses. While traditionally applied to economic or ecological systems, it is equally suitable for analyzing social mechanisms such as the decay of social trust, fluctuations in public sentiment, and governance feedback processes. Consequently, this study adopts an interdisciplinary research framework that integrates perspectives from medicine, psychology, mathematics, communication studies, and sociology. Through this integration, we develop a system dynamics model that more accurately captures the evolving characteristics of rumor propagation and governance during public health crises (44).

Inspired by the classical infectious disease SIR model, Daley and Kendall proposed the D-K compartment model, which transplants the framework of epidemic dynamics into the study of rumor diffusion (45, 46). The model is premised on the close analogy between the spread of online rumors and the transmission of infectious diseases, both of which rely on interpersonal contact to achieve “infection.” A considerable body of research has adopted the SIR model and its derivatives to characterize the dynamics of rumor propagation, while further exploring diffusion pathways and intervention strategies under varying social conditions (47).

In its conventional formulation, the SIR model partitions the population into three categories: susceptibles S , infectives I , and recovered/immune individuals R . Susceptible individuals S become infected with a given probability β through contact with infectives I , while infectives I recover and transition into the immune state R with another probability γ .


$$\left\{ \begin{array}{c} \frac{\mathrm{dS}(t)}{\mathrm{dt}}=-\mathrm{\beta S}(t)I(t)\\ \frac{\mathrm{dI}(t)}{\mathrm{dt}}=\mathrm{\beta S}(t)I(t)-\mathrm{\gamma I}(t)\\ \frac{\mathrm{dR}(t)}{\mathrm{dt}}=\mathrm{\gamma I}(t) \end{array}\right.$$

With the rapid development of social networks, numerous scholars have advanced and refined models addressing rumor diffusion, online public opinion, and collective viewpoints in digital environments (44). Nevertheless, many of these models overlook the critical role of network topology in shaping rumor dynamics, rendering them less applicable to large-scale social network analysis—an issue that was not adequately addressed until the emergence of complex network models (46). Although agent-based models can simulate propagation behaviors at the individual level, they are limited in revealing the macro-level feedback mechanisms of social trust. In contrast, system dynamics can integrate social-psychological and structural variables to depict the nonlinear relationships among trust, behavior, and feedback at the macro level (48, 49). Building upon this foundation, the present study adopts system dynamics as its analytical core and extends the classical SIR framework to develop a more comprehensive dynamical model of rumor propagation.


$$\begin{array}{c} \frac{\mathrm{dB}}{\mathrm{dt}}=\underset{\text{Rumor Spread}}{\underset{︸}{\beta \cdot S\cdot \frac{B}{N}}}-\underset{\text{Debunking}}{\underset{︸}{\delta \cdot \eta \cdot B}}-\underset{\text{Forgetting}}{\underset{︸}{\mathrm{\lambda B}}}\\ \frac{\mathrm{dS}}{\mathrm{dt}}=-\beta \cdot S\cdot \frac{B}{N}+\underset{\text{Forgetting}}{\underset{︸}{\mathrm{\lambda B}}}+\underset{\text{Reinfection}}{\underset{︸}{\mathrm{\rho I}}}\\ \frac{\mathrm{dI}}{\mathrm{dt}}=\underset{\text{Debunking}}{\underset{︸}{\delta \cdot \eta \cdot B}}-\underset{\text{Reinfection}}{\underset{︸}{\mathrm{\rho I}}} \end{array}$$

B : number of rumor believers; S : number of susceptibles; I : number of immune individuals; N : total population; β : rumor transmission rate; δ : intensity of rumor-refutation interventions; λ : forgetting rate; η : rumor-refutation efficiency factor; ρ : reinfection rate.

The central objective of this model is to employ a system dynamics approach to capture the mechanisms of rumor diffusion and governance in the context of public health. Taking social networks as the fundamental setting and embedding social trust mechanisms into the analytical framework, the model not only quantifies the speed and scale of rumor dissemination but also seeks to uncover how institutional interventions and individual attributes jointly shape the diffusion process. In contrast to traditional models that focus primarily on the flow of information, this study incorporates two additional dimensions—intervention intensity and refutation efficiency factor—in order to differentiate the effects of institutional governance and social trust restoration in rumor management. Furthermore, by introducing the parameters of forgetting rate and reinfection rate, the model can simulate the complex dynamics of information decay and repeated exposure, thereby approximating the empirical reality in which rumors recur during public health crises. Rumor propagation and the social trust system align with the core assumptions of system dynamics, including feedback, accumulation, and nonlinearity. The spread of rumors is modulated by feedback from trust levels and information transparency, with trust functioning as a stock variable that evolves over time and exerts a delayed effect on propagation intensity, thereby forming a closed-loop dynamic system (50–52). Through dynamic simulations of the interactions among these variables, the model illustrates the multiple mechanisms underlying the generation, diffusion, and dissipation of rumors. In doing so, it provides a scientific basis for risk governance and policy intervention within public health systems, while operationalizing “social trust” as a testable parameter in system dynamics modeling.

The trust–rumor feedback model developed in this study is grounded in the specific social context of public health crises, wherein the interaction between risk perception and institutional trust shapes the dynamic structure of rumor propagation. The model aims to elucidate the systemic evolution of social trust under health risk conditions. In the broader exploration of rumor governance in public health systems, understanding the diffusion of refutation information across individual networks is of critical importance. Rumor dissemination is not merely the outcome of collective behavior (53), but rather a process shaped by the reciprocal feedback and interactions among disseminators, rumors, media, and audiences (1). Many existing rumor-propagation models have not adequately incorporated social trust mechanisms into their design. By introducing social trust as a formal parameter, this study is able to shed light on the intrinsic dynamics of online rumor diffusion, particularly in ways that differ from infectious disease transmission models. Within the overarching framework of the trust–rumor relationship, we further apply topic modeling techniques to construct a probabilistic word-generation model, thereby quantifying the likelihood of specific word occurrences under a given topic.


$$P(\mathrm{wor}d_{i}|\text{topi}c_{k})=\frac{\beta_{i,k}+n_{k,i}}{\sum_{j=1}^{V}(\beta_{j,k}+n_{k,j})}$$

$P(\mathrm{wor}d_{i}|\text{topi}c_{k})$ : the probability of generating word i under topic k ; $\beta_{i,k}$ : the prior count of word i in topic k ; $n_{k,i}$ : the observed count of word i in topic k ; V : the vocabulary size, i.e., the total number of distinct words included in the model.

The purpose of this model is to integrate empirical knowledge with actual observational data to estimate the likelihood of word occurrences under a specific topic, thereby providing a rigorous quantitative tool for analyzing rumor content. Unlike methods that rely solely on frequency counts, this formulation incorporates prior counts and corresponding smoothing techniques, effectively mitigating the sparsity problems commonly encountered in textual datasets. Consequently, even in situations with limited sample sizes or uneven distributions of rumor texts, the model maintains high stability and predictive accuracy.

Furthermore, to quantify the statistical characteristics of different information sources within specific topics, a conditional probability model is proposed, enabling a more nuanced understanding of how source attributes influence the content and propagation of rumors.


$$P(\text{Sourc}e_{j}|\text{Topi}c_{k})=\frac{\sum_{i=1}^{N}I(\text{Sourc}e_{i}=j\wedge \text{Topi}c_{i}=k)}{\sum_{i=1}^{N}I(\text{Topi}c_{i}=k)}$$

$P(\text{Sourc}e_{j}|$ *T* $\mathrm{opi}c_{k})$ : the probability of a source being associated with topic k ; j : the information source under consideration; I : an indicator function that equals 1 when the condition is satisfied and 0 otherwise; N : the total number of rumor samples in the dataset.

The core task of this model is to estimate, through statistical methods, the conditional probability distribution of different sources under specific topics, thereby uncovering the structural relationship between information sources and rumor dissemination. In the context of public health crises, the credibility of information sources is closely intertwined with the dynamics of rumor diffusion. Whether originating from social media platforms, professional institutions, or ordinary citizens, differences in authority and in the underlying bases of social trust significantly influence both the acceptance of information by audiences and their willingness to retransmit it. By incorporating source probabilities into the topical dimension, this framework integrates “textual characteristics of rumors” with the “mechanisms of trust,” offering a quantitative tool for understanding the driving forces behind rumor propagation.

At the dissemination stage of rumor dynamics, in order to further examine the efficiency of trust transmission, this study introduces a dynamic model of trust transfer efficiency.


$$T_{\text{trans}}=\alpha \cdot C_{\text{cred}}\cdot \exp(-\beta \cdot D_{\text{time}})$$

$T_{\text{trans}}$ : trust transmission efficiency, quantifying the effective transfer of trust during the information dissemination process; $C_{\text{cred}}$ : source credibility, reflecting the reliability of the information provider; $D_{\text{time}}$ : time delay in rumor refutation; α,β : model parameters, which, respectively, control the effects of credibility and time delay.

The central focus of this model is to reveal the key dynamic mechanisms of trust transmission, with particular emphasis on the role of time-delay effects. Trust transmission efficiency is not solely determined by the credibility of information sources; it is also significantly constrained by the time lag in rumor refutation. Against the backdrop of the rapid spread of online rumors, the “time-delay effect” emerges as a core challenge for trust governance. By highlighting the importance of timely rumor refutation, the model quantifies trust transmission efficiency to simulate the dynamic trajectories of trust systems under varying conditions of refutation timeliness.

At the same time, public emotional responses play a critical role in rumor diffusion. To address this dimension, the study further introduces an emotional analysis model designed to capture the interplay between collective affect and rumor propagation.


$$S_{\text{final}}=((S_{\text{base}}+\alpha \cdot (N_{\mathrm{pos}}-N_{\mathrm{neg}}))+\beta_{\text{panic}}+\beta_{\text{trust}}-0.5)\times 2$$

$S_{\text{final}}$ represents the final sentiment score, which indicates the overall tendency of public emotions, ranging from (0,2), with higher values reflecting more positive affective orientations. $S_{\text{base}}$ denotes the baseline sentiment score generated using the SnowNLP tool, providing an initial evaluation of the emotional polarity of the text. $N_{\mathrm{pos}}$ and $N_{\mathrm{neg}}$ correspond to the number of positive and negative keywords identified in the text, respectively. These counts are derived through dictionary-based keyword matching and frequency analysis, thereby reflecting the emotional composition of the discourse. α serves as an adjustment factor, designed to balance the influence of disparities in positive and negative keyword frequencies on the sentiment score. $\beta_{\text{panic}}$ and $\beta_{\text{trust}}$ capture the adjustment effects of panic-related and trust-related keywords, respectively, on the sentiment score.

The purpose of this model is to quantitatively assess public emotional responses in the diffusion of online health rumors and to further reveal the dynamic relationship between emotion and social trust. Beyond considering the baseline sentiment polarity of texts, the model introduces weighted adjustments for positive, negative, panic, and trust-related keywords, enabling a more precise reflection of public perceptions and attitudes toward specific rumor information. “Trust hijacking” is driven by multiple factors. During public health events, the temporary erosion of institutional trust creates space for trust to shift toward informal information sources. Emotions such as fear, anxiety, and anger amplify individuals’ demand for alternative explanations, making trust more susceptible to emotionally charged narratives. Simultaneously, social media algorithmic recommendations and echo chamber effects amplify specific narratives, causing information to be misperceived as originating from credible groups.

To further quantify the dynamic regularities of rumors across their complete life cycle—from emergence to dissipation—this study employs the following key formula of the rumor life cycle:


$$\begin{array}{l} t_{e}=\min\{t|R(t)>0.1\times R_{\max}\},\;\;t_{p}=\arg\max_{t}R(t),\\ \;t_{d}=\min\{t>t_{p}|R(t)<0.7\times R_{\max}\},\;\\ \;t_{r}=\min\{t>t_{d}|R(t)<0.2\times R_{\max}\} \end{array}$$

$$\Delta t_{e}=t_{p}-t_{e},\;\;\Delta t_{g}=t_{d}-t_{p},\;\;\Delta t_{d}=t_{r}-t_{d},\;\;\Delta t_{t}=t_{r}-t_{e}$$

$$r_{\mathrm{eg}}=\frac{R(t_{p})-R(t_{e})}{t_{p}-t_{e}},\;\;r_{\mathrm{gd}}=\frac{R(t_{d})-R(t_{p})}{t_{d}-t_{p}},\;\;r_{\mathrm{dr}}=\frac{R(t_{r})-R(t_{d})}{t_{r}-t_{d}}$$

This set of formulas quantitatively characterizes the life cycle of online health rumors, enabling the identification of critical temporal nodes and stage-specific features in the diffusion process. $t_{e}$ denotes the emergence time of a rumor, defined as the moment when rumor intensity R(t) first exceeds 10% of the maximum intensity $R_{\max}$ , marking the onset of rumor dissemination. $t_{p}$ represents the time of peak propagation, when rumor intensity reaches its maximum value. $t_{d}$ and $t_{r}$ correspond to the onset of decline and the onset of dissipation, respectively. $\Delta t_{e},\Delta t_{g},\Delta t_{d},\Delta t_{t}$ indicates the duration of each stage, including emergence-to-peak, peak-to-decline, decline-to-dissipation, as well as the total life cycle duration. The calculation of these intervals is crucial for assessing the timeliness of rumor diffusion and the responsiveness of interventions.

In addition, $r_{\mathrm{eg}},r_{\mathrm{gd}},r_{\mathrm{dr}}$ represents the transition rates of propagation, measuring the rate of diffusion during three phases: from emergence to growth, from growth to decline, and from decline to dissipation. These transition rates provide insight into both the speed of rumor spread and the intensity of societal reactions, thereby offering a quantitative basis for designing responsive strategies. The cost of rumor governance increases nonlinearly over time and as social trust erodes. Proactive management of rumors is therefore critical, because once public trust has been weakened, the resources required to restore trust—even after debunking information is issued—are substantially higher than those needed during early intervention stages.

To further analyze the role of trust in mediating rumor dynamics and to describe the coupled processes of trust and rumor dissemination, this study develops a dynamical model of trust mechanisms and rumor propagation.


$$\frac{\mathrm{dT}}{\mathrm{dt}}=\alpha (1-T)+\mathrm{\beta E}-\mathrm{\gamma RT}$$

$$\frac{\mathrm{dR}}{\mathrm{dt}}=-\mathrm{\delta R}-\mathrm{\epsilon ER}-\mathrm{\zeta TR}$$

The first equation $\frac{\mathrm{dT}}{\mathrm{dt}}$ represents the rate of change of trust, where α(1−T) describes the natural recovery of trust, βE captures the enhancing effect of external interventions on trust, and γRT reflects the negative impact of rumors on trust. $\frac{\mathrm{dR}}{\mathrm{dt}}$ models the decline of rumors, influenced by three factors: δR , ϵER , and ζTR . Here, δR represents the natural decay of rumors, while ϵER and ζTR quantify the direct suppressive effects of interventions and trust on rumor propagation, respectively. Within a system dynamics framework, these equations analyze the bidirectional coupling between trust and rumor dynamics. Trust functions not only as a background variable in social networks but also as a core determinant of both the speed and intensity of rumor diffusion. By explicitly modeling intervention effects, the framework underscores the timeliness and strategic importance of public health governance, highlighting the interactive role of trust restoration mechanisms and rumor mitigation strategies. In practical public health crises, strengthening trust-repair mechanisms, accelerating information transparency, and shortening rumor-refutation timelines can substantially reduce both the persistence of rumor dissemination and social panic.

By integrating the sentiment analysis model with quantitative indicators derived from the rumor life cycle, this study provides a comprehensive characterization of rumor propagation patterns and their impacts on social trust. Sentiment analysis captures the dynamic evolution of audience emotions, the life cycle model elucidates stage-specific features of rumor dissemination, and trust dynamics modeling clarifies the complex interactions between trust and rumors. This multidimensional analytical framework allows researchers to theoretically understand the mechanisms through which trust restoration and interventions operate, while offering practical guidance for public health authorities to implement more targeted and timely information governance.

**Figure 1:**
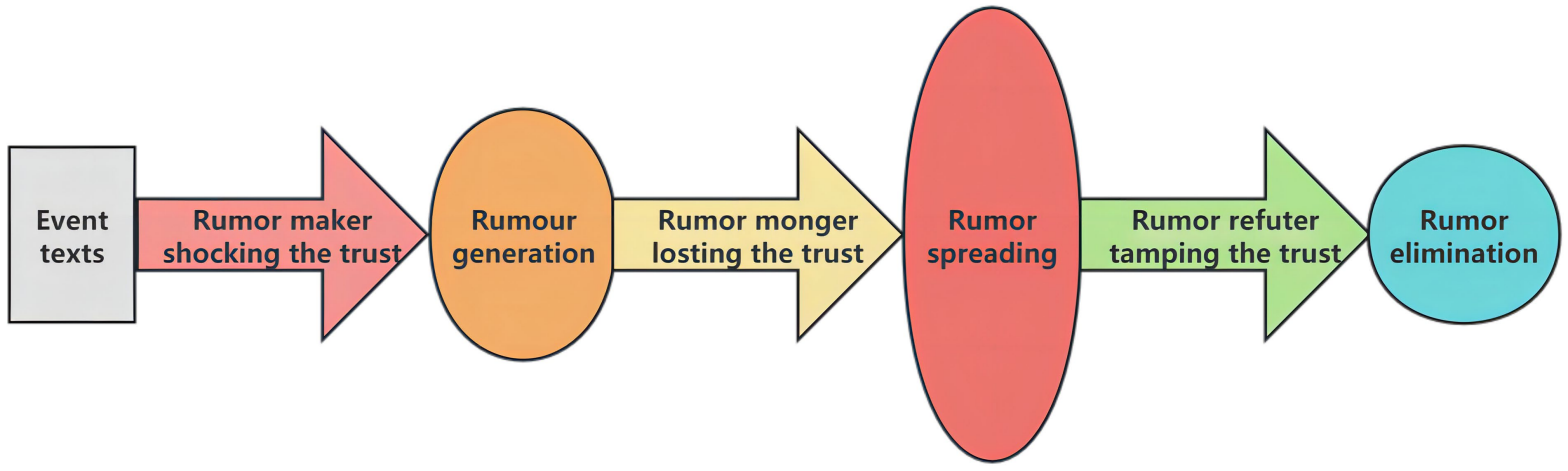
Research framework.

Throughout the analysis, this study leverages the textual content of online rumors as an empirical entry point, using specific texts to substantiate core arguments. Qualitative analysis identifies key themes and behavioral patterns, forming preliminary concepts and causal hypotheses, which are then mapped onto variables and feedback loops within the system dynamics model. Model simulations are subsequently used to evaluate the effects of different intervention scenarios. By summarizing the lexical characteristics of COVID-19-related online rumors and organizing insights based on the information conveyed in these texts, the study facilitates a shift from reactive rumor management to proactive rumor prevention in public opinion regulation (54). The overall conceptual framework of this research is illustrated in Figure 1.

## 4 The propagation cycle of health rumors and trust mechanisms

The dynamic evolution of social trust manifests not only in behavioral interactions within virtual spaces but also in the enduring structures of offline institutions and social relationships. The interplay between online and offline trust forms a systemic pathway for governing public health rumors. Online information dissemination relies on the internet as a medium and social relationships as channels for information flow. In this study, we examine the trust mechanisms of networked health rumors across three distinct stages: emergence, propagation, and dissipation.

### 4.1 Trust shocks and the emergence of networked health rumors

In the field of public health, the emergence of online health rumors is rarely a simple result of information distortion. Rather, it is closely associated with sudden disruptions in social trust. Trust shocks, as external stimuli, interact with accumulated uncertainty to facilitate the rapid emergence and dissemination of online health rumors.

#### 4.1.1 Social trust systems under sudden public events

Allport and Postman (55) identified two critical conditions for rumor formation: the significance of the event and the ambiguity of available information. Health-related events during extraordinary periods often carry a level of importance unparalleled by routine occurrences. Sudden outbreaks, such as pandemics, exert substantial shocks across industries, disrupting normal life and reshaping the trust environment. In this context, social trust systems exhibit heightened sensitivity, particularly during public health emergencies, where individuals’ reliance on information and emotional responses can accelerate the formation and spread of rumors.

Content of pandemic-related rumors is characterized by multimodality, ambiguity, and cautionary framing. Topic selection tends to prioritize proximity and practicality, narrative strategies are localized, and storytelling frameworks frequently employ fear appeals, distortions, and anchoring techniques. The high emotional arousal of call-to-action texts, the dominance of panic narratives, and the absence of negative feedback mechanisms collectively amplify perceived risks (56).

Temporally, network density, topic interaction, and structural isomorphism exhibit rapid positive lag effects on the generalization and co-variation of rumor information. Spatially, network density most strongly influences resharing behaviors, topic interactions primarily affect commenting patterns and semantic deviations, and structural isomorphism has the greatest impact on emotional polarity (57).

**Figure 2:**
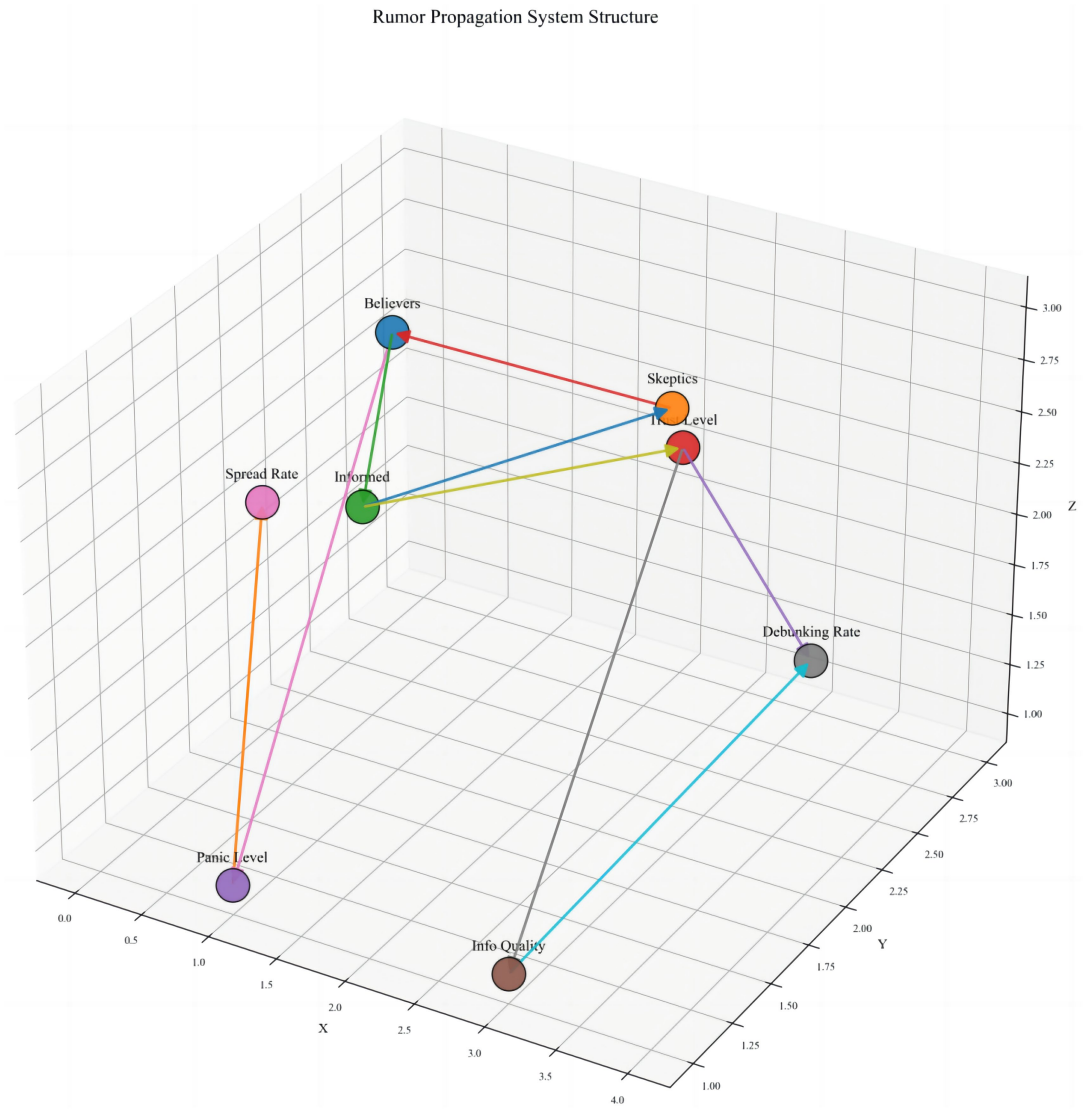
Three-dimensional structural model of the rumor propagation system in trust-related research.

The structural model of rumor propagation illustrates the complex multi-node, multi-path interaction mechanisms at a holistic level (Figure 2). The model comprises eight core variables: Believers, Skeptics, Informed, Trust Level, Spread Rate, Panic Level, Debunking Rate, and Information Quality. Directed connections between nodes indicate causal relationships. Notably, Trust Level directly affects both Skeptics and Informed individuals, highlighting its protective role in information evaluation and attitude formation. Information Quality, by enhancing the Debunking Rate, further strengthens Trust Level, creating a positive feedback loop. The Spread Rate indirectly increases the number of Believers through its effect on Panic Level, reflecting the amplifying influence of emotions on rumor dissemination. False information accelerates its spread by undermining trust networks, influencing opinion leader behavior, and altering public information-selection strategies. Compared with platforms such as Meta and X, where trust decays rapidly and information overload combined with multi-source conflicts increases rumor acceptance, Weibo’s group recommendation mechanisms help maintain relative trust stability within local communities. These mechanisms help explain cross-cultural and cross-platform differences in rumor propagation (58). Overall, this model effectively captures the interactive mechanisms among trust, information, and emotions in rumor propagation, providing a theoretical foundation for understanding the dynamic loosening of trust systems during emergent events.

From the perspective of trust mechanisms, high levels of social trust not only reduce individuals’ reliance on unverified information but also reinforce confidence in authoritative channels. Trust serves to mitigate social complexity and maintain social order (59). Regarding information mechanisms, high-quality information suppresses rumor propagation through positive feedback loops. Transparent, timely, and authoritative information dissemination enhances public trust and reduces the momentum for spreading false information (60), while corrective messages reinforce institutional credibility (61). In terms of emotional mechanisms, the coupling between Spread Rate and Panic Level demonstrates a strong association between rapid information diffusion and collective anxiety. Rapid information flows across weak-tie networks can amplify group responses and trigger chain belief effects (62). For instance, early COVID-19 rumors regarding “material shortages” led to panic buying, illustrating the interactive logic among Spread Rate, panic emotions, and the number of Believers (63).

Social trust systems are embedded within specific temporal and spatial contexts and consist of interacting agents, achieving dynamic equilibrium through the circulation of resources and information. The stability of trust systems depends on the coordinated efforts of law enforcement, public management agencies, and the general public.

#### 4.1.2 Trust logic of key actors during the rumor emergence stage

In examining the governance logic of online health rumors, information sensitivity determines how individuals respond to different sources and types of information during sudden public health crises, influencing both their receptivity and vigilance. Concurrently, trust differentiation reveals that the public tends to selectively engage with information channels and assess the veracity of information based on pre-existing social networks and trust structures. A nuanced analysis of the interaction between information sensitivity and trust differentiation is therefore essential for understanding the micro-level mechanisms and macro-level patterns of rumor propagation in public health events. Such insights provide a critical foundation for constructing more resilient social trust systems capable of mitigating the spread of misinformation.

**Figure 3:**
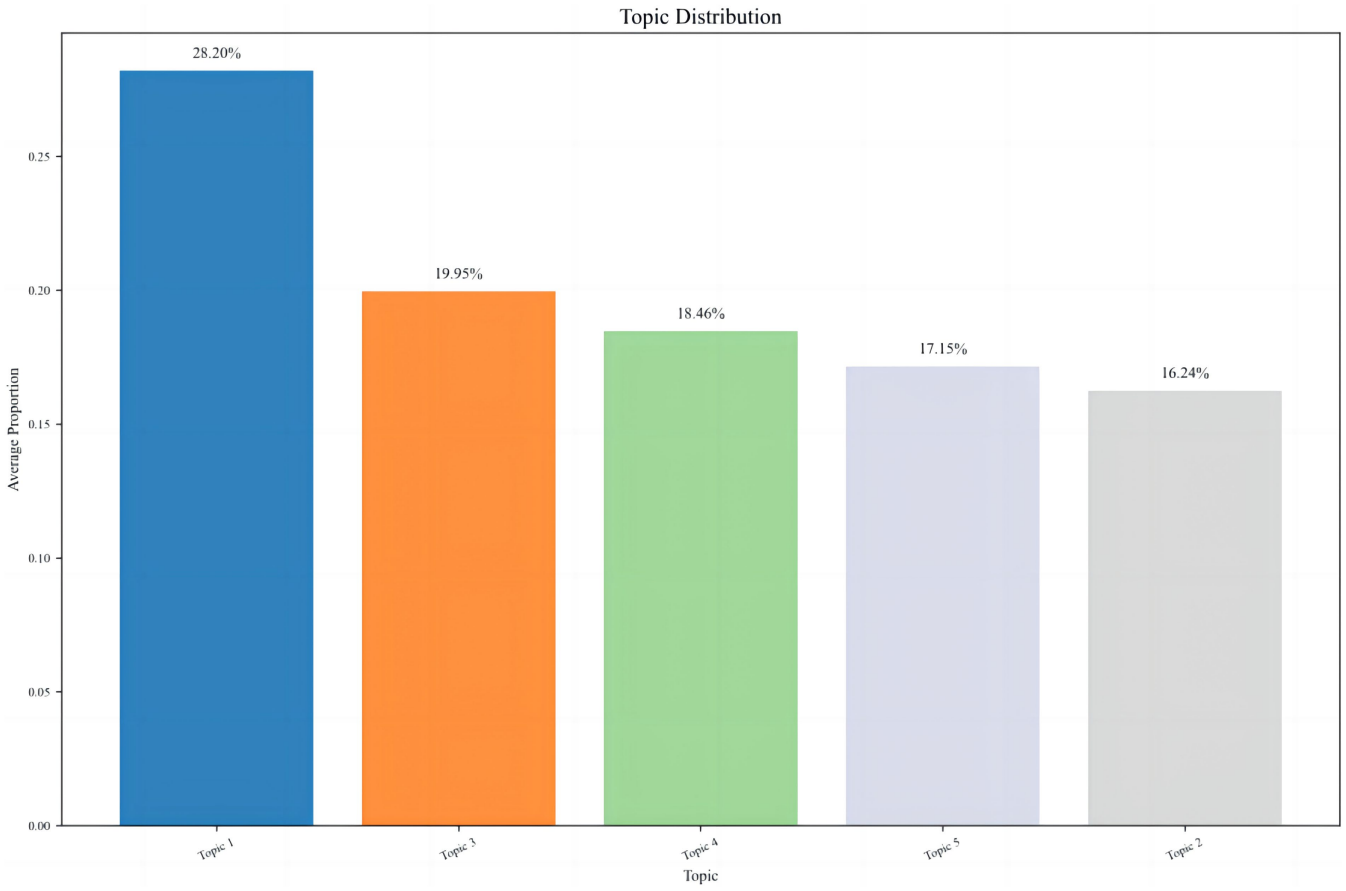
Distribution of topics related to trust in rumor discourse.

The bar chart in Figure 3 presents the average proportional distribution of five topics (Topic 1–5) derived from a Latent Dirichlet Allocation (LDA) analysis of texts related to “social trust and online rumors.” Topic 1 accounts for the largest proportion (28.20%), followed by Topic 3 (19.95%), Topic 4 (18.46%), Topic 5 (17.15%), and Topic 2 (16.24%), indicating an overall uneven thematic focus. These differences in topic proportions highlight the concentration of rumors in specific domains and the heterogeneous interactions of social trust across different areas. Topic 1, comprising nearly 30% of the corpus, corresponds to domains highly sensitive to social trust, such as public health, social governance, and public policy. In these domains, public reliance on institutional and professional trust is particularly strong. When social trust is low, the persuasive power of authoritative information diminishes, creating a “trust vacuum” that enables rapid rumor generation and dissemination. Conversely, Topics 2–5 represent areas with lower trust dependence, where the threshold for rumor propagation is higher, and even if rumors emerge, their diffusion momentum is relatively weak.

The uneven distribution of topics reveals the “trust targeting” of online rumors. Public trust anxieties in specific domains—for example, doubts regarding public policy or institutional transparency—tend to concentrate rumor activity in those areas. By integrating LDA-based keyword distribution analyses, the mechanisms by which different types of trust influence rumor propagation can be elucidated. During health crises, the public exhibits unprecedented demand for accurate epidemic information (64). The high transmissibility of COVID-19 drew widespread societal attention, producing both convergence and divergence in social trust within public spaces. While real events form the foundation of information dissemination, they are inevitably accompanied by misperceptions, and many rumors arise from intentional or unintentional misinformation. In the analysis process, this study draws on qualitative analysis of specific debunking texts to provide theoretical support and a basis for hypothesis formation, extracting core theoretical insights relevant to the model variables and feedback mechanisms. For instance, textual analysis of online health rumors shows that most contain strongly negative framing (e.g., “Residential buildings, factories, and cars caught fire due to alcohol disinfection? False”), tapping into individuals’ fears of unknown dangers.

During the pandemic, specific trust environments indirectly facilitated the emergence of online health rumors. Rumor actors often had multiple motives: some released personal frustrations as emotional venting or deliberately fabricated rumors to induce public panic (e.g., “A woman in Long’an spread epidemic rumors causing mass panic and was legally prosecuted by police”); others sought illegal profit or marketing gains (e.g., “Masks and alcohol sold at Beijing designated pharmacies on Tuesdays and Fridays” was false); and some marketing-oriented media accounts created rumors to attract attention and increase clicks (e.g., “The Jiangsu medical team’s luggage was lost” initially due to sorting errors, amplified by marketing accounts into a rumor). Each rumor may carry multiple motives, but all share a common effect: undermining trust in science, media, and other trust subsystems.

#### 4.1.3 Trust mechanisms during the rumor emergence stage

Amid the unique public health concerns of the COVID-19 pandemic, the social trust system experienced significant loosening. Rumor actors fabricated various online health rumors to match the public’s crisis expectations, while authoritative institutions, positioned at key trust nodes, could only respond after careful information verification. Interactions among different actors created a networked environment in which trust could not effectively suppress the generation of potential online health rumors. The core logic of the rumor emergence stage is that, under conditions of information asymmetry and trust gaps, audiences rapidly evaluate incoming information, and trust deficits make them more likely to accept rumors over authoritative sources.

**Figure 4:**
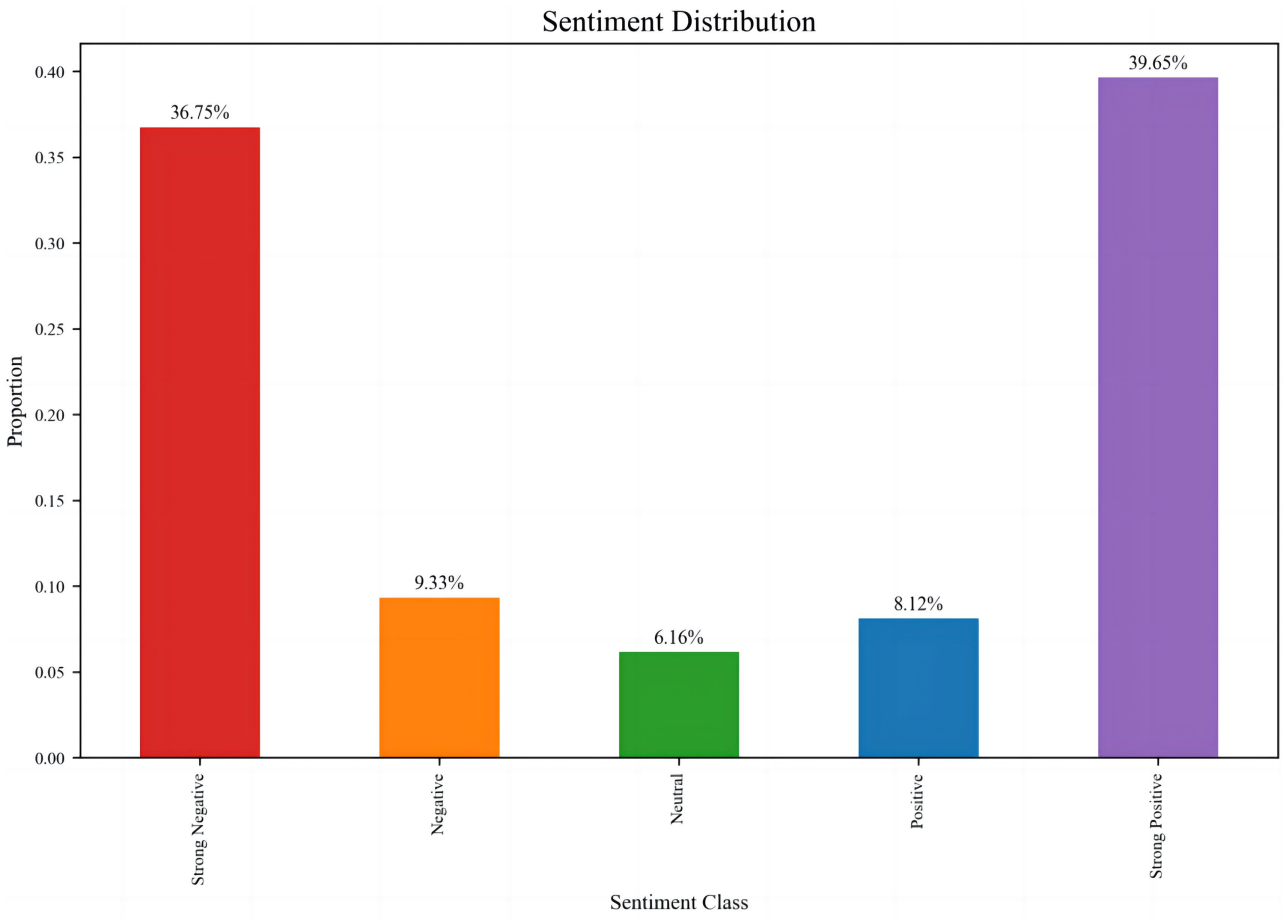
Trust-correlated topic clustering in rumor-centric text data.

The sentiment distribution of online public opinion reveals the direct impact of impaired social trust on rumor generation (Figure 4). The proportional distribution across sentiment categories is as follows: Strong Negative (36.75%), Negative (9.33%), Neutral (6.16%), Positive (8.12%), and Strong Positive (39.65%). The overall pattern demonstrates a pronounced bipolar polarization, with Strong Negative and Strong Positive sentiments dominating, while Neutral sentiment accounts for the smallest proportion, and Negative and Positive sentiments remain relatively balanced but quantitatively low. This distribution indicates a “binary extreme” emotional characteristic in online discourse, directly reflecting the dynamic pressures within the social trust environment during the rumor emergence stage.

Specifically, Strong Negative sentiment (36.75%) primarily arises from public distrust and concern regarding rumors. During the early pandemic period, widespread exposure to false information—such as the rumor “Residential buildings, factories, and cars caught fire due to alcohol disinfection?”—not only elicited public anxiety and panic but also eroded trust in public health institutions and authoritative information channels. In contrast, Strong Positive sentiment (39.65%) is mainly associated with authoritative institutions, official debunking, and the dissemination of high-quality information. Following the release of official debunking messages, the public generally responded positively, partially curbing further rumor propagation and reflecting a partial restoration of social trust and accumulation of positive sentiment. The low proportion of Neutral sentiment (6.16%) indicates that the public struggles to maintain neutrality on this issue; the construction or destruction of trust and the veracity of information readily drive polarization, creating an emotional mobilization mechanism during the rumor emergence stage. Extreme emotions spread rapidly, intensifying audience attention toward rumors and facilitating trust migration effects. The low proportions of mild Negative and Positive sentiments (9.33 and 8.12%, respectively) further suggest that moderate attitudes have limited counterbalancing effects in an environment dominated by extreme emotions. Consequently, governance strategies should prioritize leveraging Strong Positive sentiment to consolidate trust while simultaneously designing mechanisms to mitigate the trust crises triggered by Strong Negative sentiment.

The database covers topics such as epidemic prevention and control, medical resource allocation, and policy announcements. In the model, different topics correspond to distinct trust-sensitivity coefficients and rumor diffusion thresholds, reflecting variations in public trust behavior across different public health issues. In the context of public health emergencies, the social trust system exhibits marked vulnerability. Highly uncertain scenarios, such as pandemics, disrupt established social orders and lead the public to rely heavily on non-authoritative information when facing information asymmetry and ambiguity. Dynamical modeling results reveal the interactive relationship between trust levels, information quality, and emotional states. High-quality information can enhance trust by increasing the debunking rate, thereby inhibiting rumor diffusion, whereas rapid dissemination coupled with panic emotions amplifies the growth of the believer population. Trust functions both as a barrier against rumor spread and as a potential point of fragility. Collectively, the trust mechanism during the rumor emergence stage emerges as a complex product of trust gaps, emotional polarization, trust migration, and delayed authoritative responses.

### 4.2 Trust “hijacking” and online health rumor propagation

During rumor propagation, trust is often “hijacked” and transformed into a key resource for information diffusion. Individuals tend to believe messages from familiar contacts or within their own social groups, enabling rumors to leverage these appropriated trust relationships to gain greater persuasive power and dissemination capacity.

#### 4.2.1 Network Rumors under the influence of information attitudes and behavior

Once online health rumors begin to spread, rumor creators and debunkers cannot directly manipulate the trust levels of the audience. The continued propagation of a rumor largely depends on the audience’s attitudes and behaviors. Audience behavior can be categorized along two dimensions—attitude and forwarding intention—into four types: “Believe and forward,” “Disbelieve but forward,” “Believe but do not forward,” and “Disbelieve and do not forward” (Table 2). When the information is a rumor, three outcomes—“Believe and forward,” “Disbelieve but forward,” and “Believe but do not forward”—pose potential risks to the trust system, while only the group categorized as “Disbelieve and do not forward” remains fully insulated from rumor influence.

**Table 2:** Information dissemination attitude and behavior type table.

		Attitude	
		Believe	Disbelieve
Activity	Propagate	Trust and forward	Disbelieve but forward
	Non-propagate	Believe but not forward	Disbelieve and non-propagate

In the era of omnimedia, rumors leverage modern communication channels to propagate widely with novel characteristics. Networked rumors, often exhibiting group clustering, visual orientation, transience, and non-malicious intent, can spread rapidly and potentially undermine social stability. Consequently, controlling rumor dissemination and guiding public opinion have become central priorities in governance efforts (65).

**Figure 5:**
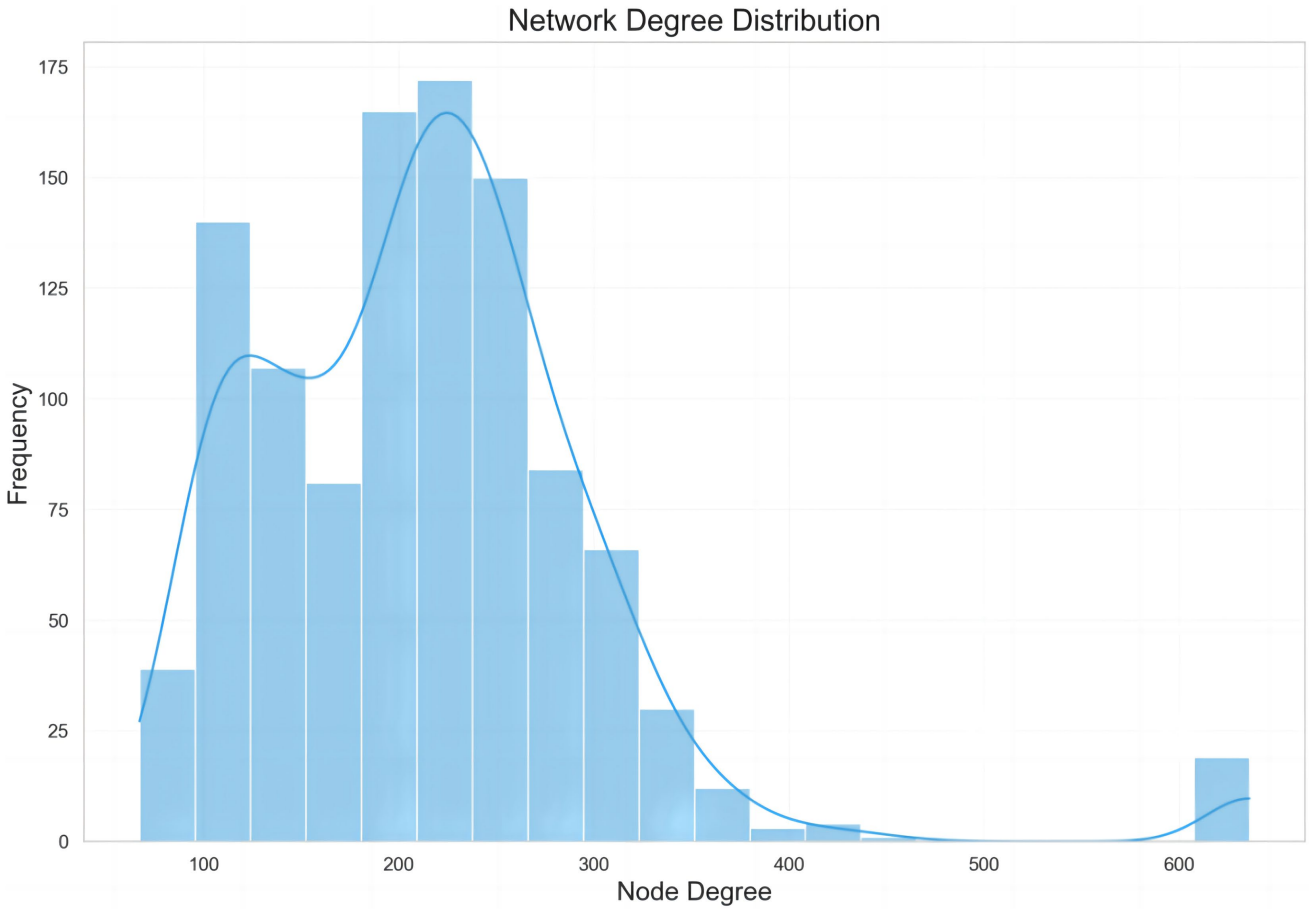
Degree distribution of nodes in rumor-trust network.

Further analysis of the network degree distribution provides structural insights into the interplay between social trust and rumor propagation networks (Figure 5). The bar-and-line graph presents the “Network Degree Distribution,” where the *x*-axis represents Node Degree (the number of connections per node) and the *y*-axis represents Frequency (the number of nodes corresponding to each degree). The results indicate that node degrees are primarily concentrated in the 100–300 range, with a peak frequency around 200–250 (approximately 175 nodes). Following this peak, the frequency declines, reaching a trough at 400–500, with a modest rebound around degree 600, although the frequency remains low. The line trend aligns with the histogram, showing a rapid initial increase, a peak, subsequent decline, and a slowly rising tail.

This distribution reflects a core–periphery network structure, where high-degree core nodes typically represent opinion leaders or high-influence accounts, acting as pivotal hubs for information dissemination and trust transmission. The sparse supernodes in the network tail (around degree 600), though limited in number, exert a leverage effect due to their broad coverage; any information disseminated by these nodes can trigger large-scale trust reconstruction or collapse. The above results differ to some extent from the diffusion patterns observed on open-network platforms such as Meta and X, where the open structure enables information to spread rapidly across groups. These platforms exhibit high node centrality and a fast yet complex propagation network. Thus, the dynamics of networked health rumor propagation are influenced not only by audience attitudes but also by the structural constraints imposed by the degree distribution of network nodes.

Integrating the propagation types in Table 2 with the network structure analysis further elucidates the micro-level mechanisms. During the pandemic, numerous internet users lacking professional health knowledge became susceptible to rumors and, under instigation by rumor spreaders, were converted into “infected” individuals. Here, the attitude of “believing” correlates strongly with trust. When confronted with information, audiences rapidly assess content based on pre-existing social trust pathways, whereas “sharing” behavior directly engages the group-level trust environment, potentially triggering cascading diffusion effects. The widespread dissemination of networked health rumors is therefore not merely a result of trust deficiency but reflects a “hijacking” of trust, whereby core nodes and trust chains are exploited. The interplay between individuals’ belief attitudes and sharing behaviors forms the driving mechanism of rumor propagation.

#### 4.2.2 Trust misrepresentation and exploitation under rumor scale effects

Networked health rumors often aim to achieve a certain scale during dissemination, reflecting an implicit individual-to-group diffusion logic. The presentation of information in the network is determined both by the choices of individual propagators and by the trust pathways embedded within social relationships. For instance, in family WeChat groups such as “All in Love as One Family,” the dissemination of health-related information often relies on fundamental kinship trust, which ensures the stable internal spread of rumors. That is, traditional trust bases are not inherently fragile but are vulnerable to misrepresentation and exploitation by rumors. In a media-saturated society, the macro-social context facilitates the proliferation of false information. The mediated dissemination of misinformation profoundly affects individuals’ cognitive uncertainty and serves as a key driver of collective emotional restructuring (8).

**Figure 6:**
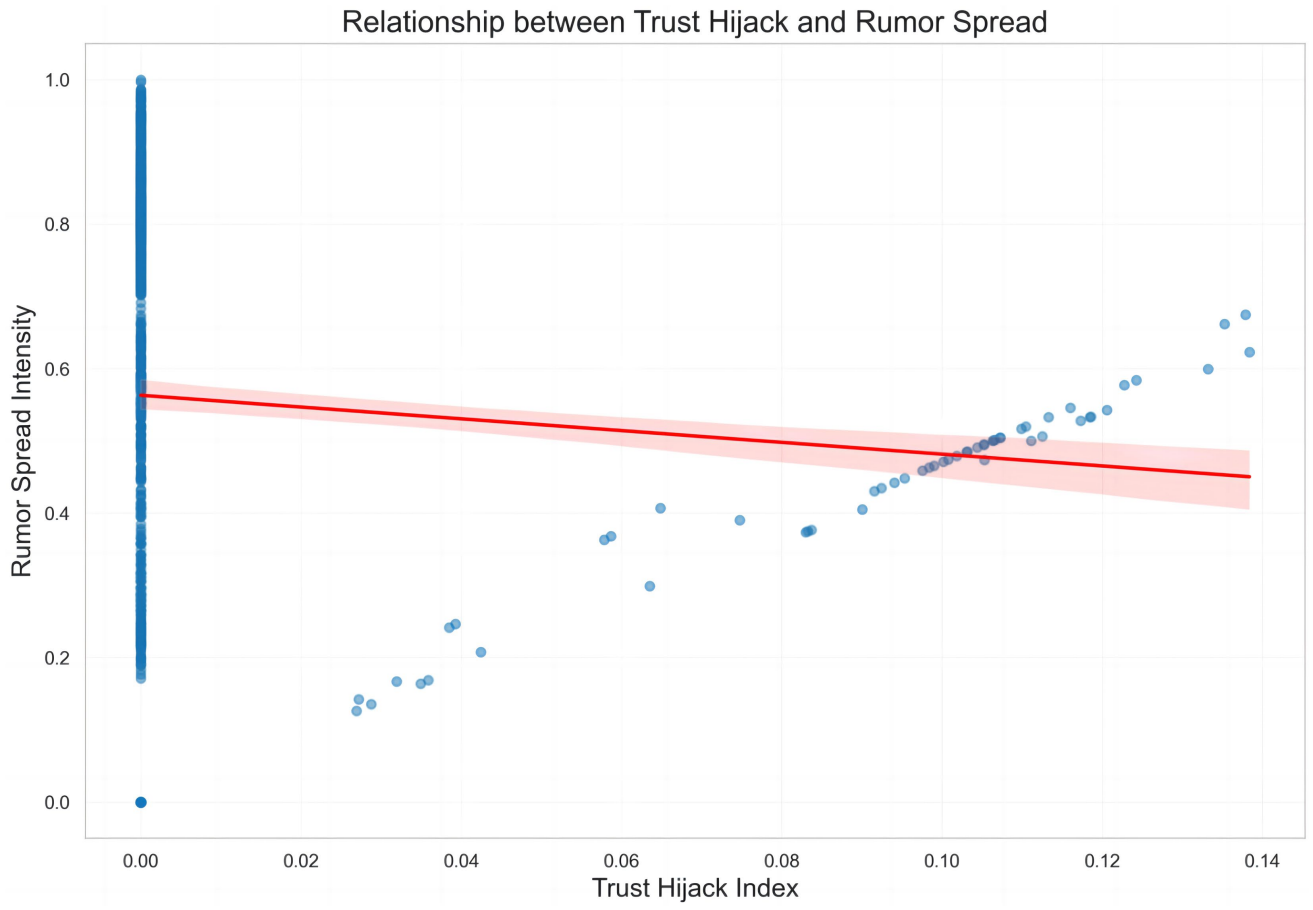
Bivariate relationship between trust hijack and rumor spread intensity.

To more clearly illustrate the scale effects of trust on rumor propagation, this study presents a scatterplot titled “Relationship between Trust Hijack and Rumor Spread” (Figure 6). The *x*-axis represents the Trust Hijack Index, while the *y*-axis corresponds to Rumor Spread Intensity. Blue data points show that when the Trust Hijack Index is near 0, rumor spread intensity is highly concentrated in the 0–1 range, forming a distinct vertical distribution band. As the index increases from 0.02 to 0.14, the points gradually disperse, with spread intensity roughly distributed between 0.2 and 0.7. The red fitted line exhibits an overall gradual decline, and the pink confidence interval covers most of the data points.

These results reveal two complex propagation logics. First, when the Trust Hijack Index approaches zero—indicating minimal trust misrepresentation or exploitation—rumor spread intensity can still reach high levels. This suggests that rumor propagation is not entirely dependent on trust hijacking but may also be driven by non-malicious factors such as individual misperception and emotional venting. Second, as the Trust Hijack Index increases, overall spread intensity tends to decrease, implying that frequent trust exploitation prompts users to activate vigilance mechanisms, actively filtering and resisting suspicious information. However, when the index exceeds 0.1, some data points show a rebound in spread intensity, highlighting a threshold effect: users may experience “trust fatigue” and lose the ability to distinguish between true and false information. Additionally, advanced deepfake techniques make impersonation more covert, triggering a new wave of dissemination. Collectively, these findings indicate that trust hijacking and exploitation exhibit a nonlinear pattern of weak negative correlation with localized rebound at the group level, suggesting that intervention strategies must precisely identify critical thresholds to prevent repeated oscillations.

“Trust hijacking” influences system equilibrium through the variable of false trust. As the trust transfer rate increases, the duration of rumor propagation extends, and the system exhibits unstable oscillations, indicating an imbalanced information ecology resulting from the hijacking of trust. At the individual level, trust hijacking primarily involves the misappropriation of authority, including scientific and media credibility, to legitimize rumor propagation. Examples include impersonating scientific trust (e.g., “Increasing home air conditioner temperature can prevent COVID-19? Rumor!”), impersonating media trust (e.g., forging screenshots of the official WeChat account “Dalian Release” to spread false information such as “Dalian City delays enterprise resumption”), and leveraging self-media with weak professional ethics to exaggerate therapeutic efficacy (e.g., claims by “bee therapy” practitioners regarding overstated media reports). These strategies allow rumors to gain passive acceptance channels within individual cognition, particularly among groups with limited critical capacity, facilitating further sharing and diffusion (17). Accordingly, trust hijacking constitutes a key driving force in the individual-level propagation of rumors.

**Figure 7:**
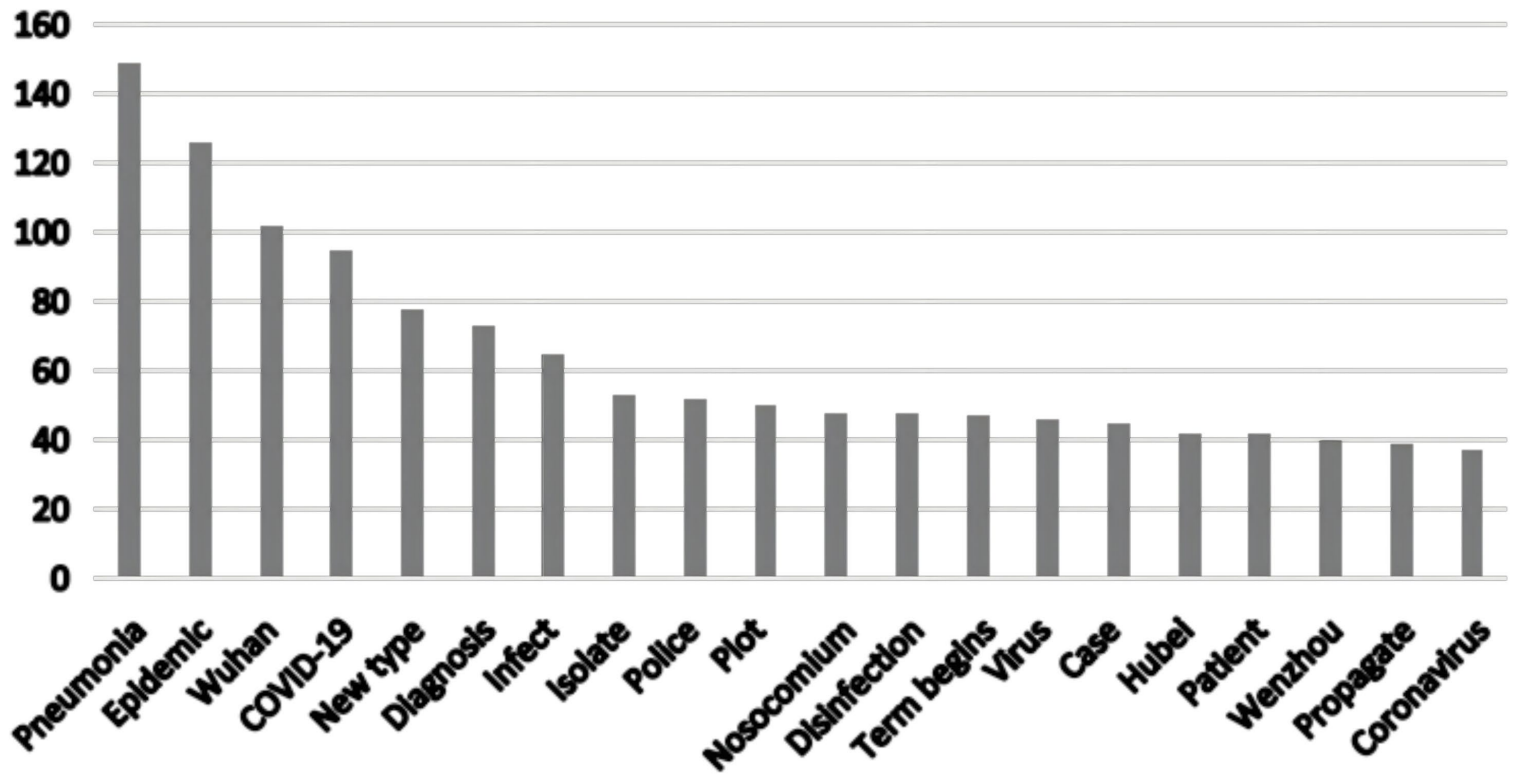
Frequency chart of the top 20 words in refuting rumors.

At the group level, rumor exploitation of trust manifests through mechanisms of leveling, sharpening, and assimilation (55), which further consolidate the rumor’s perceived legitimacy within the group, ultimately achieving cognitive control (66). Analysis of debunking texts (Figure 7) reveals that the term “pneumonia” appears most frequently, reflecting widespread attention to pandemic-related information. Interestingly, “school reopening,” though not directly related to the virus itself, ranks 13th due to anxieties among teachers, parents, and students. This pattern indicates that rumors do not diffuse uniformly but instead leverage group-specific trust pathways to penetrate and amplify their influence.

Further examination shows that this trust exploitation extends beyond kinship trust (e.g., “these messages circulated among family groups are all rumors!”) to peer trust (e.g., “School reopening in Hefei on March 2? Hefei Education Bureau debunks!”), neighbor trust (e.g., “Reports of suspected pneumonia patients in Yibin’s Nanxi Sunshine Community are rumors”), and even generalized trust within social media networks, such as WeChat groups and friend circles, where information is packaged as “urgent notices” (e.g., a forwarded “urgent notice” later confirmed by relevant authorities as a rumor). These cases demonstrate that rumors can utilize stratified trust (“differential trust”) to achieve cross-layer, large-scale propagation within social networks.

It is important to note that rumor propagation is not always driven by malicious intent. In many instances, individuals forward messages—such as those reporting confirmed cases within a neighborhood—with good intentions, aiming to alert others rather than incite panic. However, even well-intentioned trust exploitation amplifies the group-level control effect of rumors. In the logic of “insider information,” rumors often function as a perceived scarce resource, circulating rapidly online. Given the low-cost replicability of digital information, once a rumor is endowed with a trust label, it can spread widely, ultimately resulting in systematic manipulation of group cognition.

#### 4.2.3 Trust mechanisms during the rumor propagation stage

Network health rumors can be conceptualized as a form of “trust hijacking,” analogous in logic to moral or emotional coercion, where messages propagated under the guise of “care” or “love” are difficult for recipients to resist. Drawing from the concept of moral coercion, we define trust hijacking as: “the phenomenon in which individuals leverage trust to impose excessively high or unrealistic standards, coercing or influencing others’ behaviors.” Mechanistically, rumors achieve this through trust impersonation and trust exploitation, which, respectively, confer legitimacy to content and legitimacy to dissemination pathways. This dual process allows rumors to exert cognitive control from individual to group while maintaining the appearance of an intact trust structure.

This framework aligns with the contextuality, stratification, and network embeddedness of social trust: the former shapes the legitimacy of content, while the latter reinforces the legitimacy of dissemination pathways. Their interaction facilitates the large-scale diffusion of rumors, demonstrating how trust mechanisms are strategically co-opted to maximize propagation efficiency.

**Figure 8:**
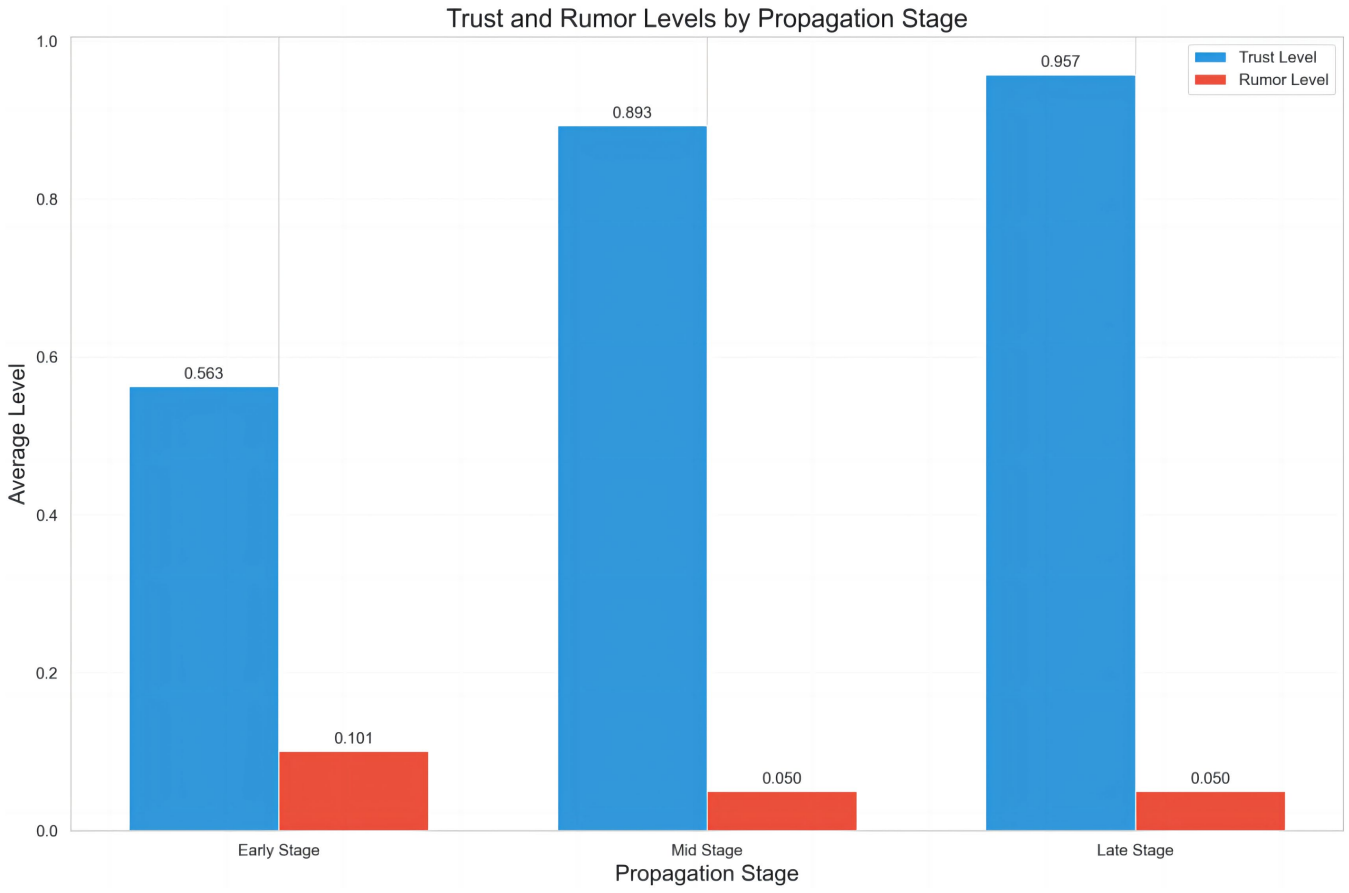
Distribution of trust and rumor levels across rumor propagation stages.

We conducted a quantitative and dynamic assessment of trust mechanisms across the rumor propagation stages (Figure 8), presented as “Trust and Rumor Levels by Propagation Stage.” The *x*-axis represents the propagation stages—Early Stage, Mid Stage, and Late Stage—while the *y*-axis indicates the average level, encompassing both Trust Level and Rumor Level. The data reveal that during the Early Stage, the trust level was 0.563 and rumor level 0.101; in the Mid Stage, trust increased to 0.893 while rumor decreased to 0.050; and in the Late Stage, trust reached 0.957, with rumor remaining at 0.050. These values indicate a progressive accumulation of trust accompanied by a decline in rumor prevalence, providing an empirical foundation for stage-specific mechanism analysis.

Regarding trust hijacking, one may ask whether rumors framed under the guise of trust can propagate unchecked. The answer is clearly no. Rumors of minor impact are often dismissed by audiences (“Can eating more ginger prevent COVID-19? No!”) without causing direct trust damage. However, when rumors lead to significant economic loss (“Stop hoarding! These drugs cannot prevent COVID-19!”), threaten life and health (“Is the Wuhan Institute of Virology leaking the virus? Rumor!”), or disrupt social order (“Citywide red lights, motor vehicle bans, total lockdown? Both notices are rumors!”), the ultimate effect extends beyond immediate harm to undermine social networks, interpersonal relations, and trust pathways. The “damage threshold” rises with contextual severity; when rumors penetrate critical situations affecting resource allocation and public order, their erosion of trust structures accumulates exponentially.

During the rumor propagation stage, the mechanisms of social trust are further highlighted through the interplay of attitude selection, relational embedding, and stage evolution. First, individual cognitive attitudes and behavioral intentions directly shape rumor diffusion dynamics. Audiences’ “believe-and-share” behaviors act as accelerators, while even skeptical but curiosity-driven or well-intentioned sharing can still compromise the trust system. Second, the scale effect of propagation reveals the dual logic of trust impersonation and trust utilization. Rumors acquire content legitimacy by impersonating scientific, media, or official authorities, while path legitimacy is ensured via kinship and neighborhood relational chains. This mechanism enables rumors to penetrate from individual to group without directly destroying trust relationships, exhibiting a nonlinear “weak negative correlation–local rebound” diffusion pattern. Third, examining stage-specific dynamics shows an inverse trajectory between trust and rumor. Initially, trust ambiguity leads to a “rumor-first, trust-lag” scenario; mid-stage interventions by authoritative debunking restore trust rapidly and compress rumor space; by the late stage, trust consolidation coexists with residual rumors, which may still reactivate under specific conditions.

### 4.3 Trust consolidation and the dissipation of online health rumors

In the process of rumor dissipation, the reconstruction and consolidation of trust are particularly critical. Only when the public reaffirms the reliability of authoritative channels can trust act as a barrier against misinformation.

#### 4.3.1 Rumor lifecycle

No rumor propagates indefinitely. Analysis of propagation outcomes indicates that online health rumors exhibit a clear lifecycle. Some rumors have limited transmission and may decay naturally (e.g., “Shanghai epidemic experts recommend setting off firecrackers to eliminate the virus? Rumor, do not believe!”), whereas others require direct intervention by authoritative institutions (e.g., “Cambodian Prime Minister Hun Sen infected? Rumor! Government officials debunk: fake news.”). For particularly persistent rumors, multiple rounds of debunking may be necessary (“Stop sharing! The image circulating in the parent group is a rumor!”). Absolute propagation periods vary significantly across rumors, reflecting the staged nature of their lifecycle.

**Figure 9:**
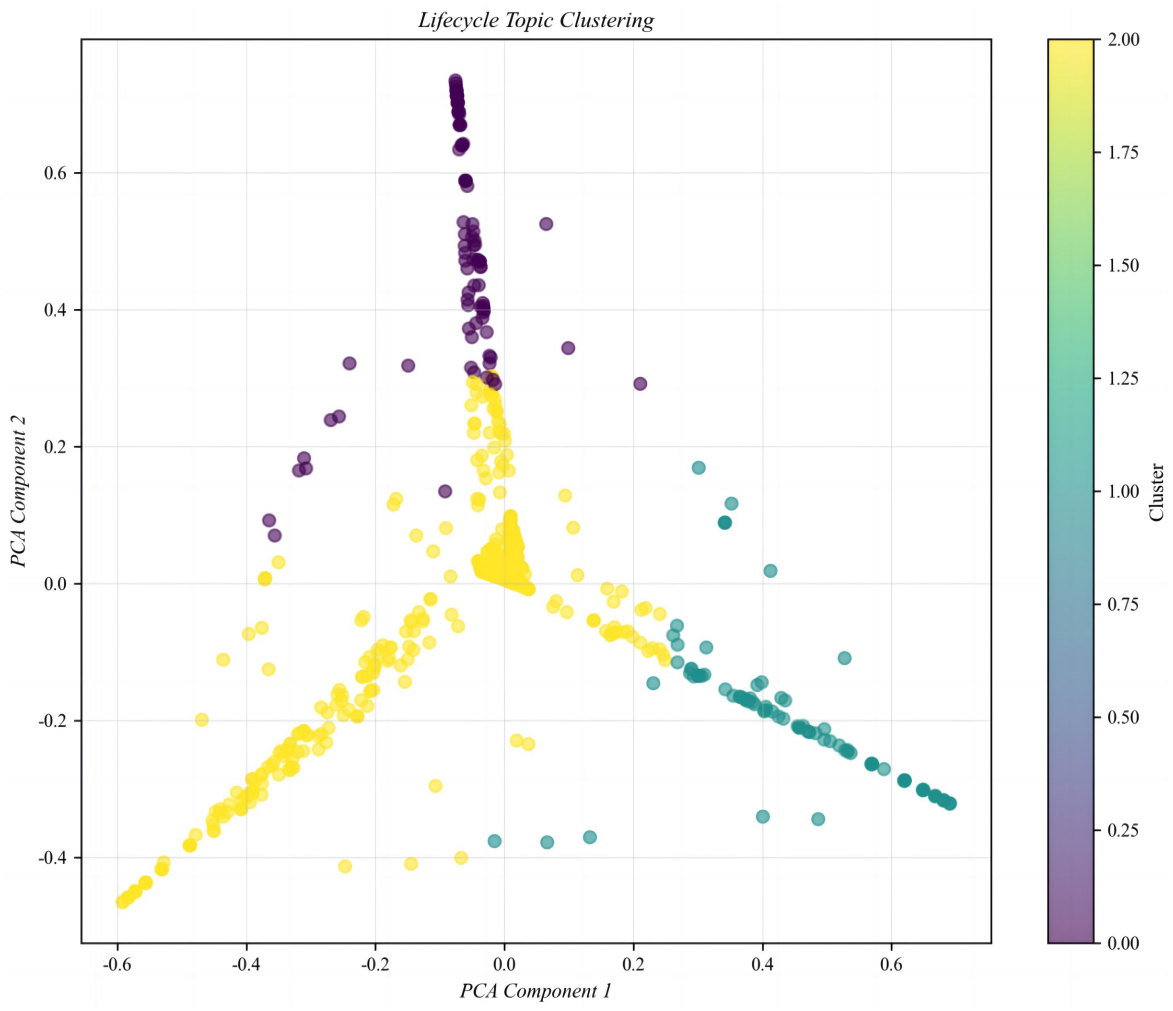
PCA-based topic clustering of rumor discourse across lifecycle in trust-related research.

Based on topic distribution characteristics across propagation stages, we conducted an analysis using lifecycle topic clustering (Figure 9). The *x*-axis represents PCA Component 1, the *y*-axis represents PCA Component 2, and the color bar on the right denotes Cluster (cluster category, range 0.00–2.00). The scatterplot demonstrates multiple clustering patterns. The purple cluster is concentrated in the Component 1 ≈ −0.2–0 and Component 2 ≈ 0.3–0.7 range; the yellow cluster predominantly occupies Component 1 ≈ −0.6–0.2 and Component 2 ≈ −0.4–0.2; while the blue-green cluster spans Component 1 ≈ 0.2–0.6 and Component 2 ≈ −0.4–0.2. Although there is partial overlap among clusters, the overall distribution exhibits relative independence, reflecting distinctive topic differentiation across propagation stages. The spatial distribution of clusters in the PCA space captures the stage-specific evolution of topics within the network rumor lifecycle.

The purple cluster (Cluster ≈ 0.00–0.25) occupies mid-to-high values in Components 1 and 2, corresponding to emerging topics in the early propagation stage. At this point, information has not yet diffused widely, and topics cluster due to novelty and low verification, aligning with the initial low-trust environment where rumors easily proliferate. The yellow cluster (Cluster ≈ 1.75–2.00) is positioned in low Component 1 and mid-to-low Component 2 ranges, representing diffusive topics during the mid-stage of propagation. Here, the dissemination scope expands, and topics aggregate due to controversy and emotion-driven engagement, consistent with mid-stage trust repair and the competition between rumors and credible information. The blue-green cluster (Cluster ≈ 0.50–1.25) covers high Component 1 and low Component 2 values, reflecting consensus topics in the late stage. After verification and screening, authoritative information dominates, and topics cluster due to legitimacy and high trust, corresponding to trust consolidation and residual rumor characteristics. Partial overlap between clusters (e.g., yellow and blue-green clusters intersecting at Component 1 ≈ 0.0–0.2, Component 2 ≈ −0.2–0.0) reveals topic interaction across propagation stages.

Although individual online health rumors have limited lifecycles, attention must be paid to the variant “reproduction” effect during propagation. Even with substantial governance resources, debunking failures may occur, where repeated corrections fail to prevent re-dissemination of previously debunked information (6). Currently, the “treat each outbreak locally” approach dominates online rumor management, which diverges from the research ideal of suppressing rumors at their source. Decentralized networks, through multi-node information sources and distributed verification mechanisms, effectively curb the spread of false information and offer multi-level pathways for trust restoration. The media plays a crucial role in rumor governance, as its fact-checking and clarification actions reduce the public’s likelihood of accepting misinformation. Accordingly, it is necessary to reconsider governance strategies by integrating social trust mechanisms, implementing precision and stage-specific interventions across lifecycles, propagation stages, and topic evolution.

#### 4.3.2 Trust-rebuilding strategies for rumor dissipation

In governing online health rumors, information debunking and administrative enforcement constitute the core strategies for rebuilding social trust. Current practices primarily rely on debunking mechanisms and deterrence policies (26). Although there are numerous enforcement cases with substantial regulatory effort, the lack of structured control mechanisms allows rumors to persist (23), indicating that coercive measures remain necessary for social stability. To improve governance of online rumors, it is crucial to examine the propagation structure of debunking information across media platforms and identify key factors influencing debunking effectiveness, thereby providing novel insights for authorities engaged in rumor mitigation (67).

**Figure 10:**
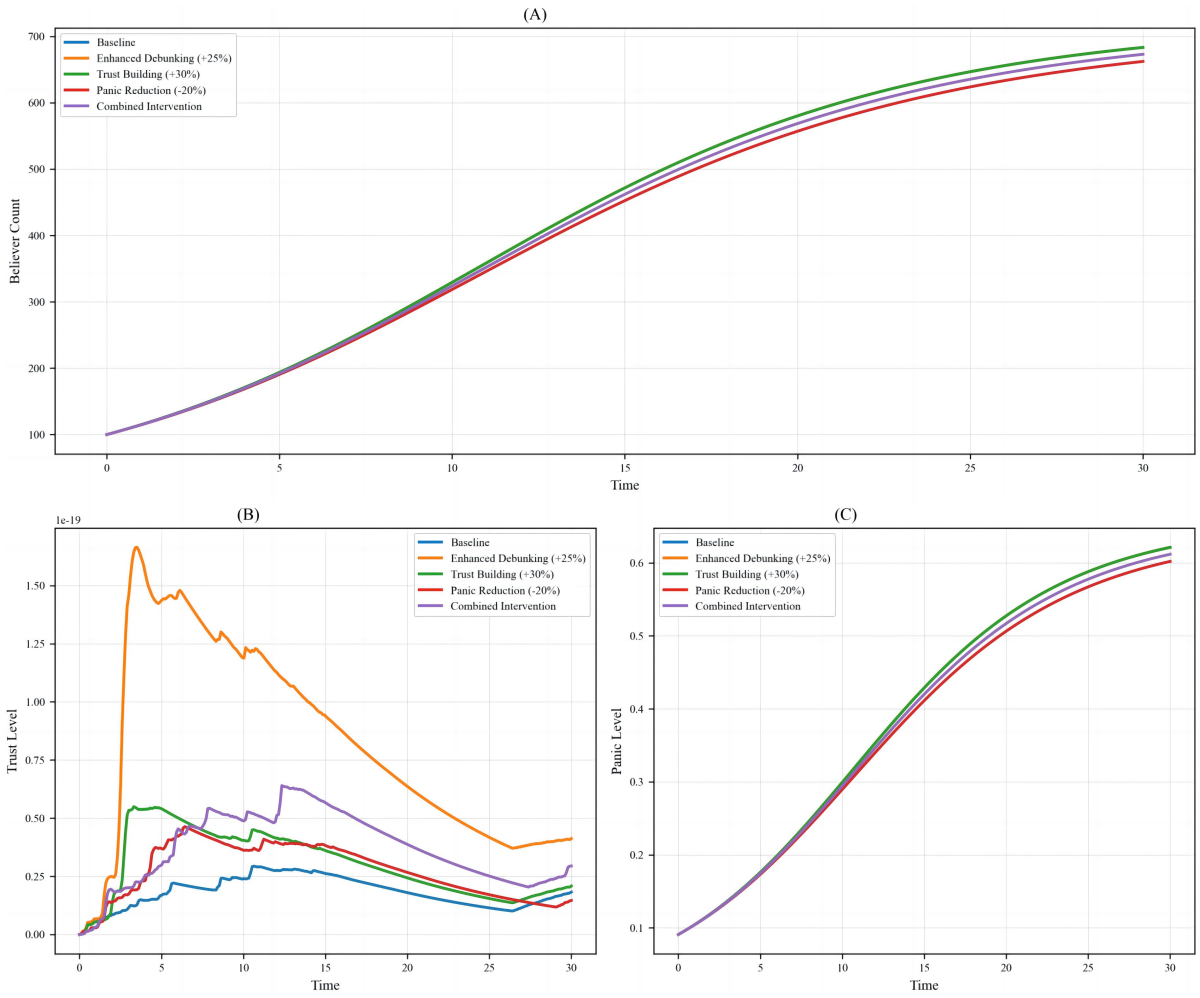
Temporal analysis of intervention strategies’ impact on believer count, trust level, and panic level in rumor propagation. **(A)** Impact on believer count. **(B)** Impact on trust level. **(C)** Impact on panic level.

Under different intervention scenarios, Figure 10A illustrates that the number of rumor believers steadily increases over time, stabilizing around periods 25–30. Compared with the baseline, scenarios of “trust enhancement (+30%)” and “panic reduction (−20%)” significantly decelerate the growth rate of rumor believers. The single intervention of “strengthened debunking (+25%)” demonstrates a relatively limited effect, whereas the “comprehensive intervention” exhibits the most pronounced suppression overall. Figure 10B presents the trajectory of trust levels: under the baseline, trust remains at a relatively low range; in the early phase of the trust enhancement scenario, trust increases rapidly but subsequently declines, whereas comprehensive intervention maintains a moderate and relatively stable level. Figure 10C depicts the evolution of panic levels, showing a gradual rise and stabilization across all scenarios. However, both panic reduction and comprehensive intervention notably mitigate the rate of increase.

These findings suggest that single-dimension interventions are limited in their capacity to curb rumor propagation and rebuild trust, and they may lack temporal effectiveness. In contrast, the comprehensive intervention, integrating debunking, trust-building, and panic management, sustains lower levels of rumor believers and stable trust over an extended period, highlighting the critical value of institutionalized governance and multi-dimensional strategies in suppressing rumor diffusion (68). Moreover, the evolution of panic levels indicates that public emotions are not merely passive variables but can reciprocally influence dissemination dynamics. Reliance solely on debunking is insufficient to alleviate panic; instead, a dual strategy of reducing panic while enhancing trust establishes a psychosocial “immune barrier” (19). Each set of simulation results presents not only numerical and curve variations but also the qualitative social implications behind them. Under multi-strategy intervention scenarios, understanding why certain combinations of interventions can rapidly restore public trust requires qualitative observation to uncover the correspondence between specific variable fluctuations and real-world social behavior patterns, thereby enhancing the explanatory power of the results. Rumor propagation is a dynamic process shaped jointly by trust, information, and emotion. Trust functions both as a protective mechanism and a potential vulnerability, necessitating reinforcement through high-quality information cycles to inhibit rumor diffusion.

From a governance perspective, these results underscore the need to reconstruct “precision-oriented logic” in trust strategies. In preemptively consolidating social trust, information transparency and public engagement play a pivotal role. Compared with a reactive approach that waits for rumors to emerge, timely dissemination of authoritative information by official agencies and media achieves preventive “immunity,” reducing rumor generation (26). Transparent information disclosure not only disseminates accurate health knowledge but also enhances the public’s understanding of their environment, thereby increasing the number of initially immune individuals in the propagation model and reducing contact rates between susceptible and infected actors (44). Public health institutions regularly release epidemic statistics, updates on medical resource allocation, and policy adjustments. By disclosing the formulation process of prevention strategies, risk assessment reports, and underlying scientific evidence, they help the public understand the logic behind decision-making. Meanwhile, feedback is collected through multiple channels such as social media, official hotlines, community bulletins, and public consultation meetings. Shifting from solely authoritative debunking to a model where authoritative information guides the public and citizens voluntarily resist rumors reinforces the social trust foundation. Timely and transparent communication from authoritative institutions, combined with the narrative control of mainstream media, equips the public to remain calm during emergent events, forming the “backbone of trust,” which prevents online health rumors from misleading the public under the guise of authority and safeguards social stability.

#### 4.3.3 Trust mechanisms in the rumor dissipation stage

Debunking represents only a holistic concept; effective rumor governance must encompass multiple strategies and approaches. Governance should follow the principles of mediated coordination, engaging government, media, platforms, and the public in collaborative oversight (56). It is essential to recognize that with the rapid innovation of information technologies such as artificial intelligence, traditional challenges in online rumor management are encountering emerging complexities, necessitating dynamic adjustments to governance logic and frameworks (69).

**Figure 11:**
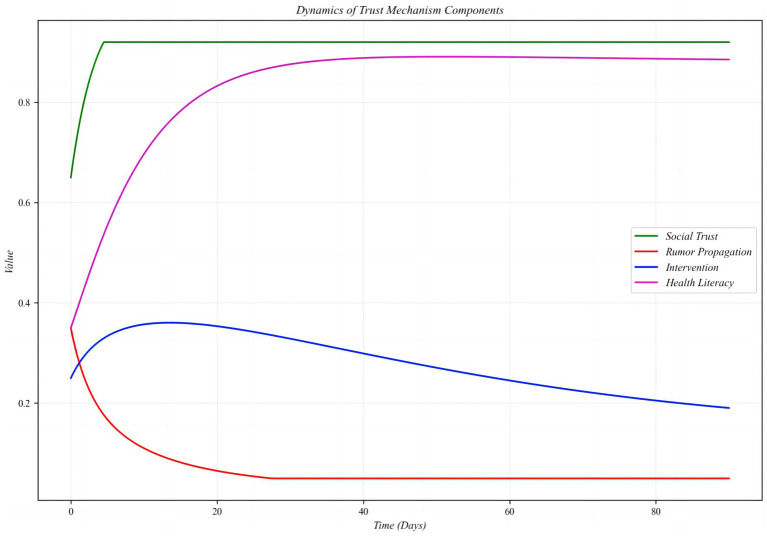
Temporal dynamics of trust mechanism components and rumor propagation.

This study conducts a temporal analysis of trust mechanisms to elucidate the dynamics of rumor propagation and intervention (Figure 11). The figure, titled “Dynamics of Trust Mechanism Components,” depicts four key trajectories: social trust (green curve), rumor propagation (red curve), intervention (blue curve), and health literacy (purple curve). Social trust starts at approximately 0.65, rising rapidly to ≈0.9 within days 0–5, after which it remains stable. Rumor propagation begins at ≈0.35 and declines sharply to ≈0.1 over days 0–20, maintaining a low and stable level thereafter. Intervention initially registers at ≈0.25, peaking at ≈0.35 within days 0–10 before gradually decreasing to ≈0.2 by day 80. Health literacy starts at ≈0.35 and steadily increases to ≈0.9 over days 0–40, subsequently sustaining a stable plateau.

Within the context of social trust and online rumor governance, social trust and rumor propagation exhibit a “see-saw” dynamic: rapid early-stage recovery of social trust coincides with a sharp decline in rumor spread, demonstrating the suppressive effect of trust restoration on rumor diffusion. Specifically, in the first 0–5 days, social trust surges from 0.65 to 0.9, while rumor levels plummet from 0.35 to below 0.1. This pattern aligns closely with the temporal window of authoritative interventions. When the social trust system is quickly restored, users’ thresholds for accepting information increase, leaving rumors with insufficient fertile ground to propagate.

Intervention measures display a “short-term activation—long-term decay” profile, peaking around days 0–10 at ≈0.35 and gradually diminishing thereafter, highlighting the temporal sensitivity of intervention strategies. Health literacy exhibits a “gradual accumulation—steady-state support” effect on trust mechanisms, progressively increasing over days 0–40 to ≈0.9 and sustaining this level, illustrating its stabilizing contribution to social trust.

Debunking typically occurs after rumors have already exerted social impact, incurring both economic and trust-related costs. Compared to preemptive trust-building and normative measures that prevent rumor emergence, *post hoc* debunking is inherently reactive. Beyond clarifying the original rumor, the propagation of derivative or distorted “variants” generated during the rumor lifecycle must also be addressed. Legal sanctions against rumor-mongers—ranging from warnings to detention—can exert a deterrent effect but simultaneously consume governance resources. Collectively, these findings suggest that timely dissemination of authoritative information prior to rumor emergence can significantly enhance social trust and reduce the subsequent costs associated with debunking (Table 3).

**Table 3:** Governance strategies of different rumor development stages.

	Before the rumor started	After the rumor spread
Social trust	Information disclosure	Explain and clarify
Legal norm	Deterrent warning	Sanction

Table 3 presents governance strategies corresponding to different stages of rumor development. Prior to rumor emergence, strategies emphasize information transparency to enhance social trust, alongside deterrent warnings to reinforce legal norms. Post-emergence interventions primarily focus on explanation, clarification, and punitive measures. Deterrence theory offers important guidance for upstream governance of online rumors, particularly in cases motivated by illegal profit-seeking behavior (26). Simultaneously, maintaining a balance between public order and freedom of expression, and delineating administrative regulatory boundaries, is critical in online rumor governance (70). In the early stage of the pandemic, the regular release of confirmed case numbers and policy updates—disseminated simultaneously through platforms such as Weibo and WeChat—effectively enhanced public trust in government information and curbed the spread of rumors. The construction of information transparency depends not only on health institutions themselves but also on the coordinated support of governments, experts, and other authoritative bodies. Institutional trust plays a crucial role in restoring virtual trust. When offline social trust structures remain stable, the peak intensity of online rumor propagation decreases significantly, confirming the systemic role of offline trust in the governance of public health rumors.

During the rumor dissipation stage, online health rumors exhibit a dynamic lifecycle. Different rumors demonstrate stage-specific evolution: some decay naturally, while others require authoritative clarification, sometimes repeatedly. Mainstream news media need to continuously disseminate officially verified information to curb the spread of rumors. The synergistic interaction between media intervention and decentralized network structures can systematically suppress rumor diffusion and enhance the public’s capacity to restore trust. Lifecycle topic clustering indicates that early-stage topics cluster around novelty and low verification, mid-stage topics spread due to emotional salience and controversy, and late-stage topics converge on authority and consensus. Dynamic analysis of trust mechanisms further elucidates the “see-saw” logic: rapid social trust restoration compresses the space for rumor propagation, while long-term accumulation of health literacy provides sustained stabilization of trust. Overall, the central task during rumor dissipation is trust reconstruction, encompassing both repair of trust fractures caused by rumors and reinforcement of public trust through information transparency, legal deterrence, and health literacy enhancement. This approach reduces debunking costs, prevents the resurgence of dormant rumors, and supports the long-term resilience of the public health system.

## 5 Discussion

The findings of this study indicate that online health rumors in public health contexts are not isolated instances of false information flow, but rather complex social processes deeply intertwined with social trust mechanisms. System dynamics modeling reveals the differential role of social trust across the generation, propagation, and dissipation stages of rumors. In the generation phase, trust deficits provide fertile ground for rumor emergence; during propagation, trust impersonation and trust exploitation allow rumors to circumvent rational skepticism; and in the dissipation phase, reconstruction of trust becomes the key driver for information clarification and the restoration of collective cognition. The findings of this study are consistent with those of Vosoughi et al. (19), who demonstrated that false information spreads faster, farther, and more broadly than true information within online networks, and that the speed of rumor diffusion is significantly influenced by the level of social trust. The results also align with Lewandowsky et al. (42), showing that in both Western and Chinese contexts, the erosion of trust accelerates the dissemination of misinformation, thereby confirming the central role of social trust in shaping information diffusion. These findings not only address the practical challenges posed by the trust–rumor interaction but also provide a novel explanatory framework for understanding information ecologies during public crises. Cinelli et al. (63) pointed out that COVID-19–related rumors exhibit high transmissibility and complex diffusion structures across social media platforms. Investigating the causes, characteristics, and governance challenges of online rumor propagation holds significant practical value for developing effective regulatory strategies for digital misinformation (4).

At the theoretical level, this study advances the dynamic understanding of social trust. Traditionally, trust has been treated as a static form of social capital or institutional resource; here, we emphasize its temporal evolution. First, this study clarifies the mediating role of trust in rumor propagation and demonstrates its cross-platform applicability. Second, it proposes and validates the theoretical mechanisms of trust hijacking and trust recovery, confirming their operational feasibility through model simulations and qualitative analysis. Third, it compares the effectiveness of governance strategies across different social media ecosystems, providing empirical support for the proposed theoretical framework.

Studies by Lewandowsky et al. (60) and Nyhan et al. (71) both indicate that correcting false information requires a sustained process, and the effectiveness of interventions is moderated by trust. Social trust thus plays a central role in the process of information correction (60, 71). By employing system dynamics methods, we demonstrate both the centrality and the fragility of trust in social integration, providing empirical support for understanding trust evolution in the digital era. The study further integrates key causal loops with existing theoretical literature. Yin Fei et al. (29) applied system dynamics modeling to rumor governance in emergencies, highlighting media influence and the degree of online polarization as critical factors affecting rumor propagation. Li Shizheng et al. (30), through a rumor evolution model in mobile social networks, demonstrated that user psychology and platform content moderation significantly influence rumor popularity. Consequently, enhancing information credibility and user trust emerges as a central pathway for curbing rumor diffusion.

At the practical level, the findings offer multiple insights for rumor governance in public health systems. First, governance strategies should not rely solely on *post hoc* information clarification or technical debunking but must proactively construct and maintain trust relationships. Second, during rumor propagation, channels of trust impersonation—such as false expert identities or algorithmically amplified false content—should be identified and mitigated. Finally, in the rumor dissipation stage, strategies should prioritize social-psychological repair, using community engagement and opinion-leader guidance to rebuild trust. The uniqueness of public health crises is reflected not only in information uncertainty but also in the multi-layered mechanisms for restoring social trust. A loss of public trust in the health system manifests in the widespread anxiety over personal and collective safety, rather than merely economic loss. Moreover, the heightened time sensitivity of public health crises renders social trust a critical variable that must be restored within a short timeframe.

Even though rumor propagation follows a certain lifecycle, timely and proactive governance remains essential. First, rumor diffusion is time-sensitive, and interventions must occur before critical cognitive inflection points; otherwise, the marginal effect of information correction declines rapidly. Second, the restoration of social trust exhibits a temporal lag: if institutions fail to respond during the early stages of rumor spread, public trust recovers slowly even when clarifications are intensified later. Third, delayed intervention increases both direct governance costs—such as information dissemination, platform moderation, and public opinion monitoring—and indirect costs, including social–emotional and psychological interventions.

During governance, attention must also be paid to the potential influence of sociodemographic variables on rumor propagation and trust dynamics. The heterogeneity of different age groups, occupational categories, and online-active communities may lead to significant differences in information diffusion speed and trust recovery time. Simultaneously, legal or administrative regulations should clarify processes for information disclosure, data collection, and publication to ensure institutional enforcement. Relevant agencies should collaborate with higher-level authorities, expert committees, and community organizations to maintain consistency and scientific rigor in information, preventing interdepartmental conflicts from undermining the effectiveness of transparency measures.

Authoritative media involvement in fact-checking, combined with transparent and timely information release mechanisms, strengthens public trust. Meanwhile, social media platforms should enhance self-regulation, cross-platform fact-checking, and the integration of multi-node information sources to reduce the risk of rapid rumor propagation within centralized networks. Further, it is necessary to integrate the characteristics of international social platforms to develop a unified theoretical framework for rumor governance. This framework should encompass information source attributes, network structures, user behaviors, and the dynamics of social trust, thereby revealing both the common mechanisms of rumor propagation and intervention as well as platform-specific differences, and enabling the dynamic adjustment of governance strategies.

This study has several limitations. First, although the system dynamics model effectively captures the macro-level interactions between trust and rumor propagation, consistent with previous research demonstrating that system dynamics can simulate overall dissemination dynamics and intervention effects in the study of misinformation and rumors (51), it remains limited in revealing the micro-level psychological drivers of individuals and the detailed structures of social networks. Second, the data relied upon in this study are primarily drawn from publicly available sources, with samples skewed toward highly active users. These data may be constrained by temporal, spatial, and platform-specific factors, making full generalization to other contexts challenging. Moreover, qualitative analyses inherently involve subjectivity in sample selection and text coding; however, cross-source data validation, coding consistency checks, and theoretical triangulation help ensure the interpretive robustness of the results. Third, while this study emphasizes the importance of social trust, structural differences in trust across cultures or institutional contexts may limit the model’s applicability. Although cross-platform analysis is incorporated, the model is still constrained by data accessibility and platform privacy policies, particularly regarding user behavior patterns and algorithmic recommendation mechanisms on international platforms.

By embedding social trust into the core framework of public health rumor governance through a system dynamics approach, this study provides a foundational analysis. Future research could extend these insights across interdisciplinary and cross-cultural contexts, offering a more comprehensive understanding of, and response to, the challenges of rumor governance in the digital age.

## 6 Conclusion

Analysis of early-stage COVID-19 online health rumors reveals that during public emergencies, the public’s sensitivity to health information is highly susceptible to manipulation, undermining the social trust foundation. Online health rumors exploit preexisting trust pathways to “hijack” public trust, highlighting the critical role of information transparency and civic participation in governance. Systematic analysis across rumor generation, propagation, and dissipation demonstrates the centrality of social trust in rumor dynamics and informs trust-based governance strategies.

During the generation stage, social trust exhibits a duality: trust deficits provide psychological and institutional space for rumor emergence, while high-quality information and authoritative responses exert early-stage suppression. Factors such as sensitivity to health risks, differences in institutional and professional trust, emotional amplification, and selective information reception collectively shape the complex logic of rumor generation, indicating the necessity of trust management within public health systems. During the propagation stage, micro-level trust cognition and macro-level network structures are tightly coupled, producing a nonlinear “trust impersonation–trust exploitation” diffusion mechanism. Individual “belief–forwarding” behaviors are influenced not only by information veracity but also by social embeddedness and stage-specific emotional fluctuations, driving rumors from individual to collective scale. Governance strategies must account for both audience discernment and trust dynamics to establish resilient social trust barriers.

In the dissipation stage, trust reconstruction is central. The rumor lifecycle exhibits stage-specific evolution, requiring differentiated interventions. Sole reliance on reactive debunking or enforcement may induce short-term governance fatigue, whereas precise, coordinated strategies combined with legal trust frameworks can simultaneously compress rumor space and build long-term social trust. Focusing on “trust hijacking” holds significant implications for public health governance. In rumor management, public health institutions must not only implement mechanisms for correcting misinformation but also actively manage the redistribution of trust. By establishing a “preemptive trust mechanism,” institutions can engage in open communication during the early stages of governance to prevent trust from shifting toward non-authoritative information sources. Public health rumor governance should thus transition from passive *post hoc* debunking to proactive rumor prevention.

While recognizing the harms of online health rumors, it is also important to acknowledge potential opportunities. Rumors can function as a “safety valve” for non-institutionalized political participation in China’s risk society (72). Effective trust-based rumor governance strategies involve authoritative official communication, timely updates, and public engagement in a more inclusive and participatory manner (26). The effectiveness of public health governance depends not only on the robustness of information technologies and institutional transparency but also on the coordinated construction of social trust across virtual and real-world spaces. System dynamics simulations indicate that, under limited resources, governance efforts should be prioritized during the early stages of rumor propagation to maximize marginal effectiveness. At the same time, the heterogeneity of social groups must be considered. Information interventions and public opinion monitoring should incorporate demographic characteristics such as age, occupation, and education level to design differentiated strategies and enhance governance efficiency.

Future research can be expanded in several directions. First, integrating individual-level experimental and survey data can strengthen the model’s micro-level explanatory power. Second, conducting cross-platform and cross-national comparisons can reveal how trust–rumor interaction mechanisms vary across different institutional contexts. Third, exploring the synergistic mechanisms between technological and social governance can further extend the application of system dynamics in rumor research, including integration with multi-agent modeling and micro-level calibration of trust variables, thereby enhancing the model’s ability to account for individual behavioral heterogeneity and diverse propagation pathways and achieve more comprehensive governance outcomes. Through these expansions, public health systems can develop a more operational and scientifically grounded framework for managing online rumors.

## Data Availability

The raw data supporting the conclusions of this article will be made available by the authors, without undue reservation.
